# Systemic Inflammation and Colorectal Cancer: Assessing the Role of Reverse Causality

**DOI:** 10.3390/cancers18193190

**Published:** 2026-10-02

**Authors:** Fatemeh Safizadeh, Tafirenyika Gwenzi, Marko Mandic, Michael Hoffmeister, Hermann Brenner

**Affiliations:** 1 Cancer Prevention Graduate School, German Cancer Research Center (DKFZ), 69120 Heidelberg, Germany; fatemeh.safizadeh@dkfz-heidelberg.de (F.S.); marko.mandic@dkfz-heidelberg.de (M.M.); 2 Division of Clinical Epidemiology of Early Cancer Detection, German Cancer Research Center (DKFZ), 69120 Heidelberg, Germany; tafirenyika.gwenzi@dkfz-heidelberg.de (T.G.); m.hoffmeister@dkfz-heidelberg.de (M.H.)

**Keywords:** systemic inflammation, inflammatory biomarkers, colorectal cancer, C-reactive protein

## Abstract

**Background/Objectives**: Prospective studies have reported associations between systemic inflammatory biomarkers and colorectal cancer (CRC) risk, but whether these reflect a causal role of inflammation or responses to preclinical disease remains uncertain. **Methods**: Using data from 383,850 UK Biobank participants (aged 40–69 years; recruited 2006–2010), we investigated potential reverse causality by assessing these associations according to time since biomarker assessment. Baseline systemic inflammatory markers, including C-reactive protein (CRP), albumin, and blood cell-derived ratios, were assessed. Multivariable Cox models estimated associations with CRC risk across predefined follow-up intervals. **Results**: Over a median follow-up of 11.8 years, 4996 participants developed CRC. Inflammatory biomarkers were associated with increased CRC risk, particularly during the early follow-up. Associations weakened substantially or disappeared when early follow-up was excluded. For example, CRP was associated with a 3.03-fold higher CRC risk (highest vs. lowest quartile; 95% CI: 2.06–4.45) within the 0–1-year interval, while no association was observed during the 3–5, 6–8, or >8-year intervals. Across complete follow-up, the association remained statistically significant but much weaker (HR: 1.17; 95% CI: 1.07–1.28). Similar patterns were observed for most other biomarkers, except the platelet-to-neutrophil ratio. **Conclusions**: Our results suggest that the associations between inflammatory biomarkers and CRC risk may partly reflect reverse causality arising from preclinical disease.

## Simple Summary

Systemic inflammation has been associated with colorectal cancer (CRC), but it remains unclear whether elevated inflammatory biomarkers contribute to CRC development or reflect, at least partly, inflammation caused by undiagnosed cancer. In this study, we investigated the associations between nine circulating inflammatory biomarkers and CRC risk in the UK Biobank, with particular attention to the time between biomarker assessment and CRC diagnosis. We found that associations were strongest among participants diagnosed shortly after recruitment and generally weakened with increasing follow-up time, with a similar temporal pattern observed across different biomarker measures. These findings suggest that early associations may partly reflect reverse causality, whereby undiagnosed CRC contributes to elevated systemic inflammation. Examining associations according to time since biomarker assessment may therefore provide important insights into the relationship between systemic inflammation and CRC risk.

## 1. Introduction

Inflammation has been implicated in carcinogenesis through multiple biological mechanisms [1]. Epidemiological studies have reported associations between circulating inflammatory biomarkers and the incidence of cancer overall as well as several site-specific cancers, including CRC [2,3,4]. In light of these findings, inflammatory biomarkers have also been explored as potential tools for the early detection of CRC [5,6,7].

Evidence indicates that chronic systemic inflammation induced by environmental and lifestyle factors, such as tobacco smoking, inhaled pollutants, obesity, and dietary factors, can contribute to tumorigenesis [8]. At the tissue level, CRC develops within a complex inflammatory tumor microenvironment in which malignant epithelial cells interact with immune cells, including macrophages, neutrophils, and lymphocytes, and with stromal components such as cancer-associated fibroblasts and endothelial cells [9,10]. Through cytokines, chemokines, and reactive oxygen and nitrogen species, these cellular interactions may promote DNA damage, tumor-cell proliferation, angiogenesis, immune evasion, and metastasis [8,9,10]. These local interactions can also evoke systemic responses through the release of inflammatory mediators that affect distant organs, including the liver and bone marrow [10,11]. Systemic inflammation may therefore be reflected by circulating acute-phase proteins, such as C-reactive protein (CRP), and by blood cell counts or ratios, although these measures do not directly characterize the local inflammatory cell infiltrate within colorectal tissue [11].

Although a causal role of systemic inflammation in carcinogenesis is plausible, the associations observed in cohort studies may also reflect, at least in part, reverse causality, i.e, systemic inflammation induced by cancers which were already present but yet undiagnosed at baseline. One way to address potential reverse causality is to examine the associations between systemic inflammatory markers and cancer risk across defined intervals of follow-up, particularly during the period shortly after baseline and over longer-term follow-up. 

We followed this approach in analyzing the association between various systemic inflammatory biomarkers and CRC risk, using data from the large UK Biobank cohort. We selected biomarkers reflecting inflammatory and immune processes potentially involved in CRC development and progression, including commonly studied biomarkers such as CRP and several other markers frequently assessed in the context of CRC prognosis [12,13,14], including neutrophil-to-lymphocyte ratio (NLR), platelet-to-lymphocyte ratio (PLR), platelet-to-neutrophil ratio (PNR), lymphocyte-to-monocyte ratio (LMR), systemic immune-inflammation index (SII), neutrophil-monocyte-to-lymphocyte ratio (NMLR), systemic inflammation response index (SIRI), and serum albumin.

## 2. Materials and Methods

### 2.1. Study Population

The design and data collection of participants of the UK Biobank have been described in detail elsewhere [15]. Briefly, more than half a million study participants aged 40–69 were recruited from England, Scotland, and Wales from 2006 to 2010. The baseline [16] assessment included comprehensive health and lifestyle information collected through self-completed touchscreen questionnaires and computer-assisted interviews, as well as physical examinations and laboratory measurements, including serum biomarkers such as CRP and blood cell counts such as neutrophils, platelets, lymphocytes, and monocytes. For this analysis, we excluded participants with a history of cancer (except non-melanoma skin cancer) and those with missing baseline biomarker data, including CRP, NLR, PLR, PNR, LMR, SII, NMLR, SIRI, and albumin.

The UK Biobank was approved by the North West Multi-centre Research Ethics Committee (MREC) as a Research Tissue Bank (RTB) (renewed approval in 2021:21/NW/0157). Written informed consent was obtained from all UK Biobank participants. The present study was conducted under UK Biobank Application No. 66591 and was approved by the Ethics Committee of the Medical Faculty of Heidelberg University (approval No. S-817/2022). Data analysis for the present study began in August 2023.

### 2.2. Biomarkers and Blood Cell Counts

A range of key biomarkers were measured in blood samples collected from all UK Biobank participants at recruitment. CRP was measured by immunoturbidimetric—high sensitivity analysis on a Beckman Coulter AU5800 (Beckman Coulter, Inc., Brea, CA, USA) in mg/L. Albumin was measured by BCG analysis on a Beckman Coulter AU5800 in g/L.

Hematological assays were also conducted at recruitment on whole blood samples from all UK Biobank participants. As part of these assays, lymphocytes, monocytes, neutrophils, and platelets were measured and reported in 10^9^ cells/L.

We derived the following inflammatory biomarkers from the blood cell counts measured at baseline: NLR = neutrophils/lymphocytes, PLR = platelets/lymphocytes, PNR = platelets/neutrophils, LMR = lymphocytes/monocytes, SII = (platelet×neutrophils)/lymphocytes [16], NMLR = (neutrophils+monocytes)/lymphocytes [17], and SIRI = neutrophils×monocytes/lymphocytes [18].

### 2.3. Colorectal Cancer Incidence

Follow-up with respect to cancer incidence was performed by record linkage with cancer registries (up to December 2020 for England, November 2021 for Scotland, December 2016 for Wales). CRC included cancers of the colon (C18.0–18.9), rectosigmoid junction (C19), and rectum (C20), and was identified using the 10th revision of the International Statistical Classification of Diseases (ICD-10).

### 2.4. Statistical Analysis

The baseline characteristics of the cohort were summarized using descriptive statistics. Person-years of follow-up were calculated from the date of recruitment (i.e., the date of blood collection) to the date of CRC diagnosis, death, loss to follow-up, or end of follow-up (country-specific end of cancer registry follow-up), whichever occurred first. Time since recruitment served as the underlying time scale. 

Cox proportional hazards models were applied to assess the associations between inflammatory biomarkers and CRC risk. CRP was considered as the primary biomarker of interest. Accordingly, the primary analysis evaluated the association between baseline CRP and incident CRC. The remaining inflammatory biomarkers were evaluated as complementary analyses to assess the consistency of the observed temporal patterns across different markers of systemic inflammation. Fully adjusted models incorporated established CRC risk factors selected a priori, including age at recruitment (years), sex (male, female), body mass index (BMI) (kg/m^2^), ethnicity (White, other), Townsend deprivation index, educational qualifications (higher academic/professional, lower academic/vocational, none), physical activity (low, medium, high) assessed using the International Physical Activity Questionnaire (IPAQ) groups [19], alcohol consumption categorized as never, special occasions only, 1-3 times a month, once or twice a week, 3-4 times a week, and daily or almost daily, pack-years of smoking (years), intake of vegetables (tablespoons/day, including raw and cooked), fruit (pieces/day, including fresh and dried), fish (<2 times a week, ≥2 times a week, including oily and non-oily), whole and refined grains (servings/week), and red and processed meat (never, less than once a week, once a week, ≥2 times a week), history of CRC screening (including faecal occult blood testing, sigmoidoscopy, or colonoscopy), history of CRC in a first-degree relative, and regular intake (self-reported prescribed) of non-steroidal anti-inflammatory drugs (NSAIDs) including aspirin. All covariates were assessed at baseline.

We estimated adjusted hazard ratios (HRs) and 95% confidence intervals (CIs) for CRC risk based on categorical biomarker values. For the primary analyses, we restricted the analytical cohort to participants with complete data for all nine biomarkers, thereby ensuring a common analytical population across all biomarker analyses. Biomarker quartiles were defined once in the total analytical cohort, with the same quartile cut-offs applied to men and women. HRs were then calculated across quartiles for each biomarker, with the lowest quartile serving as the reference category. For PNR, LMR, and albumin, which were inversely associated with CRC risk, the highest quartile of the original biomarker values was used as the reference category so that higher HRs corresponded to higher risk. To explore potential reverse causality, separate Cox models were fitted for (i) individual years of follow-up and (ii) cumulative years of follow-up. Due to the limited number of CRC cases at individual follow-up years, follow-up time was combined into four intervals: 0–2, 3–5, 6–8, and >8 years. Interval-specific analyses were conducted using delayed-entry Cox models, with each interval representing a separate period of follow-up. Restricted cubic spline analyses were additionally performed over the complete follow-up period for the natural log-transformed values of CRP, albumin, NLR, and SII to assess the shape of the continuous exposure-response associations and potential non-linearity. Three knots were placed at the 25th, 50th, and 75th percentiles of each biomarker distribution, with the median value used as the reference value. The spline models were adjusted for the same covariates as the primary analyses, and statistical evidence for non-linearity was assessed using a Wald test. 

To further explore the distribution of biomarker levels according to proximity to CRC diagnosis, we performed an additional descriptive analysis among participants who developed CRC. Log-transformed baseline biomarker values were visualized using boxplots, comparing participants diagnosed within 0–3 months of recruitment with those diagnosed ≥3 months after recruitment. Differences in biomarker distributions between the two groups were assessed using the Wilcoxon rank-sum test. This analysis aimed to descriptively characterize biomarker levels according to proximity to CRC diagnosis. 

We additionally conducted sensitivity analyses to assess the robustness of the findings. First, we repeated the analyses after excluding participants with extreme biomarker values, defined as values below Q1 − 1.5 × IQR or above Q3 + 1.5 × IQR (where Q1 and Q3 denote the first and third quartiles and IQR the interquartile range). Second, to assess the potential impact of selection arising from the requirement for complete data across all biomarkers, we repeated the quartile-based analysis of the association between CRP and CRC risk using a CRP-specific complete-case population, including all participants with available CRP measurements irrespective of missing values for the other biomarkers. We additionally investigated the associations of the natural logarithm of CRP and of a 1-SD increase in the natural logarithm of CRP with CRC risk, and assessed the quartile-based associations separately by sex and cancer site (colon and rectal cancer). Effect modification by sex was evaluated using a ln (CRP)-by-sex interaction term. Differences between CRC sites were assessed using a test for heterogeneity (case-case analysis). These analyses were conducted for both cumulative follow-up periods (0–2, 0–5, and 0–8 years, and complete follow-up) and mutually exclusive follow-up intervals (0–2, 3–5, 6–8, and >8 years). Third, for the primary biomarker, CRP, we repeated the analyses after excluding participants with prevalent inflammatory bowel disease (IBD) at recruitment. Fourth, to assess the potential influence of competing mortality, we fitted Fine–Gray [20] subdistribution hazard models for CRP in the CRP-specific analytical population across the cumulative follow-up periods (0–1, 0–2, 0–5, 0–8 years, and complete follow-up), treating death from any cause before CRC diagnosis as a competing event. The models were adjusted for the same covariates as the primary analyses, and associations were reported as subdistribution hazard ratios (SHRs) with 95% CIs.

Missing covariate data were handled using multiple imputation with the PROC MI procedure, generating five imputed datasets separately for each biomarker analysis. The imputation model included all covariates used in the corresponding multivariable analysis, as well as the respective biomarker exposure for each analysis. These were subsequently combined using Rubin’s rules with PROC MIANALYZE. The number and percentage of missing values for each covariate is shown in Supplementary Table S1. All analyses were conducted using SAS version 9.4 (SAS Institute Inc., Cary, NC, USA). *p*-values were two-sided, and considered significant below 0.05. Given the multiple analyses performed, individual *p*-values were not interpreted in isolation; the magnitude and precision of the associations and the consistency of the observed patterns across follow-up periods and biomarkers were also taken into account.

## 3. Results

**Figure 1:**
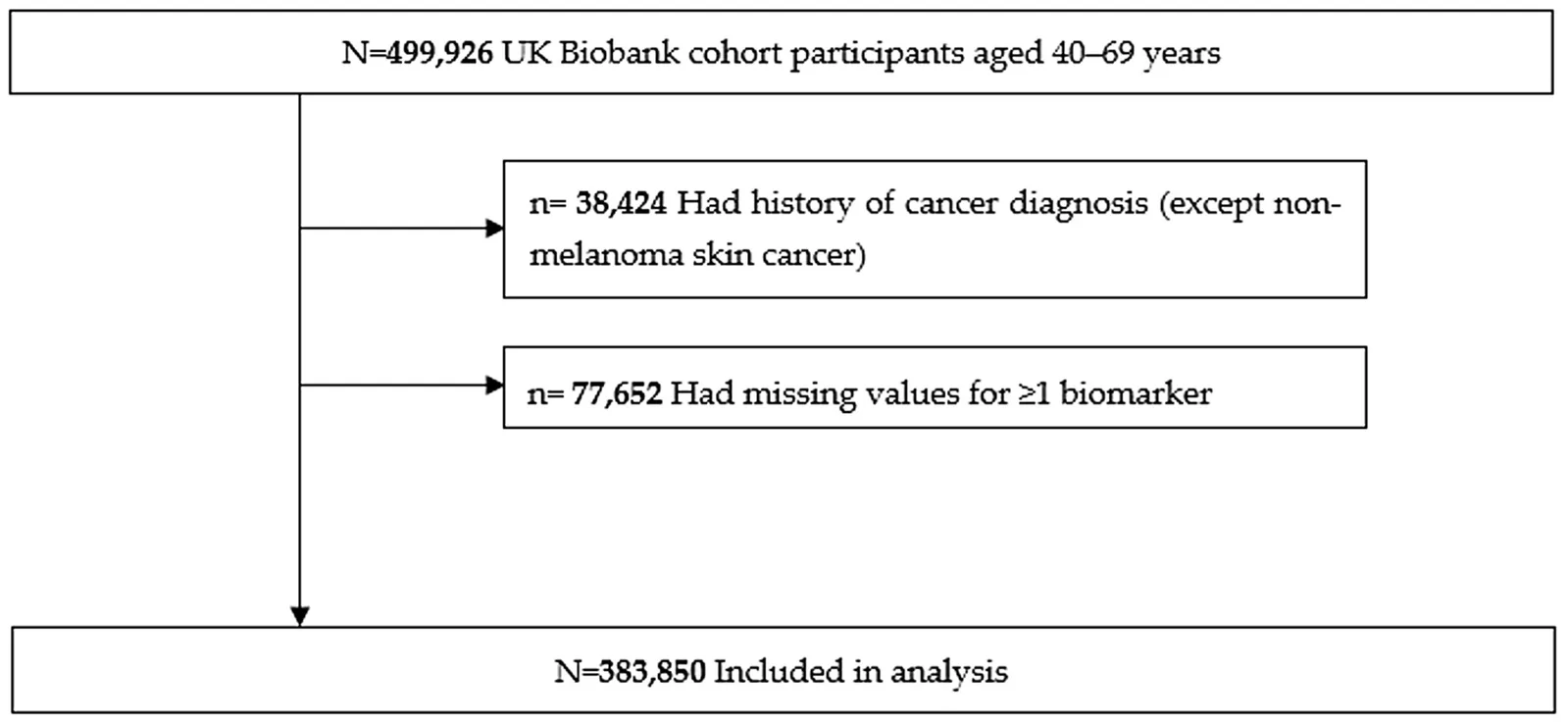
Study population flow diagram.

After excluding 38,424 participants with previous cancer diagnosis (excluding non-melanoma skin cancer) at or before recruitment, and 77,652 participants with missing values for any of the biomarkers, 383,850 participants remained for the analysis (Figure 1). Among those excluded due to biomarker missingness, 53.4% had missing values for more than one biomarker, including 22,606 (29.1%) participants with missing values for 7–9 biomarkers. Participants with complete biomarker data were comparable to those with missing biomarker data with respect to baseline characteristics, including age, sex, ethnicity, and recruitment country. Although some comparisons were statistically significant due to the large sample size, the absolute differences were small. However, the distribution of assay/acquisition year differed between the groups (Supplementary Table S2). Median follow-up time was 11.8 (interquartile range: 10.9–12.5) years, during which 4996 participants were diagnosed with CRC. Of the included participants, 88.4% were from England, 7.3% from Scotland, and 4.4% from Wales.

Table 1 presents the baseline characteristics of the study population, overall and among participants who developed CRC. Individuals with CRC were older at baseline, with a median age of 62 years, compared to 57 years in the overall cohort. Women made up 52.6% of the total population but only 42.5% of CRC cases. The majority of participants (94.5%) were of White ethnicity (British, Irish, or any other White background), while the remaining participants were of mixed, Asian or Asian British, Black or Black British, Chinese, or other ethnic backgrounds. Median BMI at baseline was slightly higher in CRC cases than in the overall cohort (27.3 kg/m^2^ vs. 26.8 kg/m^2^). The median CRP level was 1.5 mg/L among participants who developed CRC, compared with 1.3 mg/L in the overall cohort; overall, 22.4% of participants had CRP levels > 3 mg/L, while 4.0% and 1.1% had levels > 10 and >20 mg/L, respectively.

**Figure 2:**
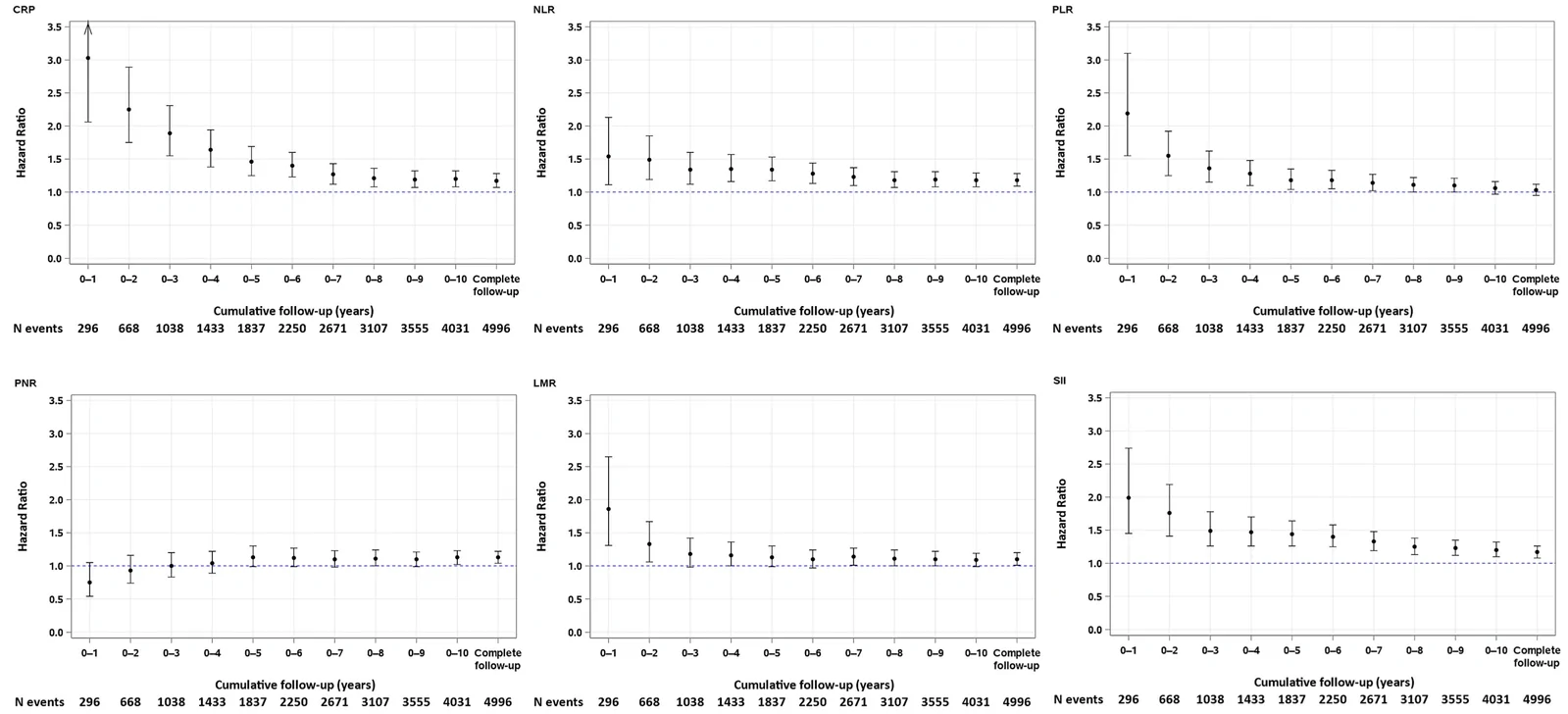

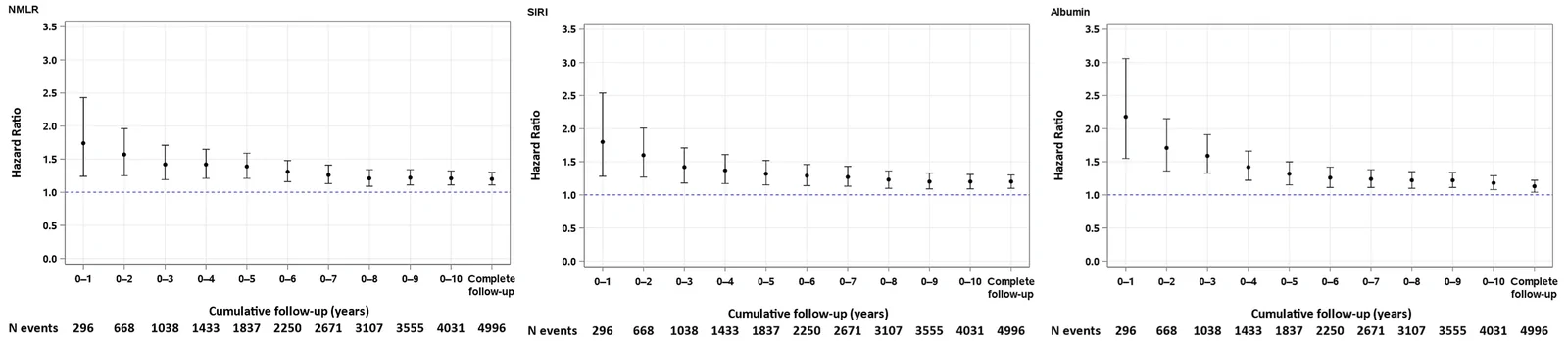
Hazard ratios (95% confidence intervals) of colorectal cancer risk for highest versus lowest quartile of various biomarkers (for PNR, LMR, and albumin, the direction of comparison is reversed; lowest versus highest quartile), for cumulative follow-up periods. Abbreviations: CRP: C-reactive protein; LMR: Lymphocyte-to-monocyte ratio; NMLR: Neutrophil-monocyte-to-lymphocyte ratio; NLR: Neutrophil-to-lymphocyte ratio; PLR: Platelet-to-lymphocyte ratio; PNR: Platelet-to-neutrophil ratio; SIRI: Systemic inflammation response index; SII: Systemic immune-inflammation index.

**Figure 3:**
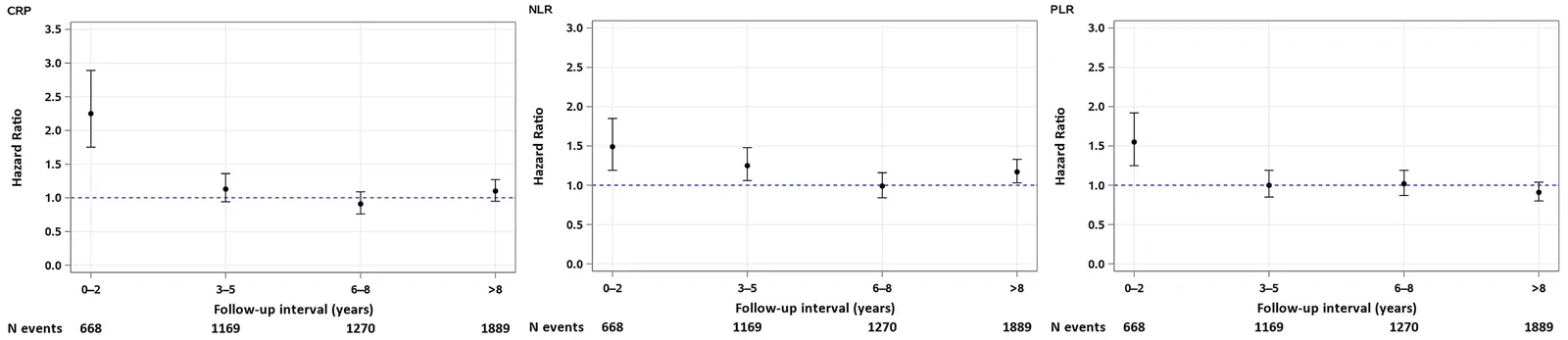

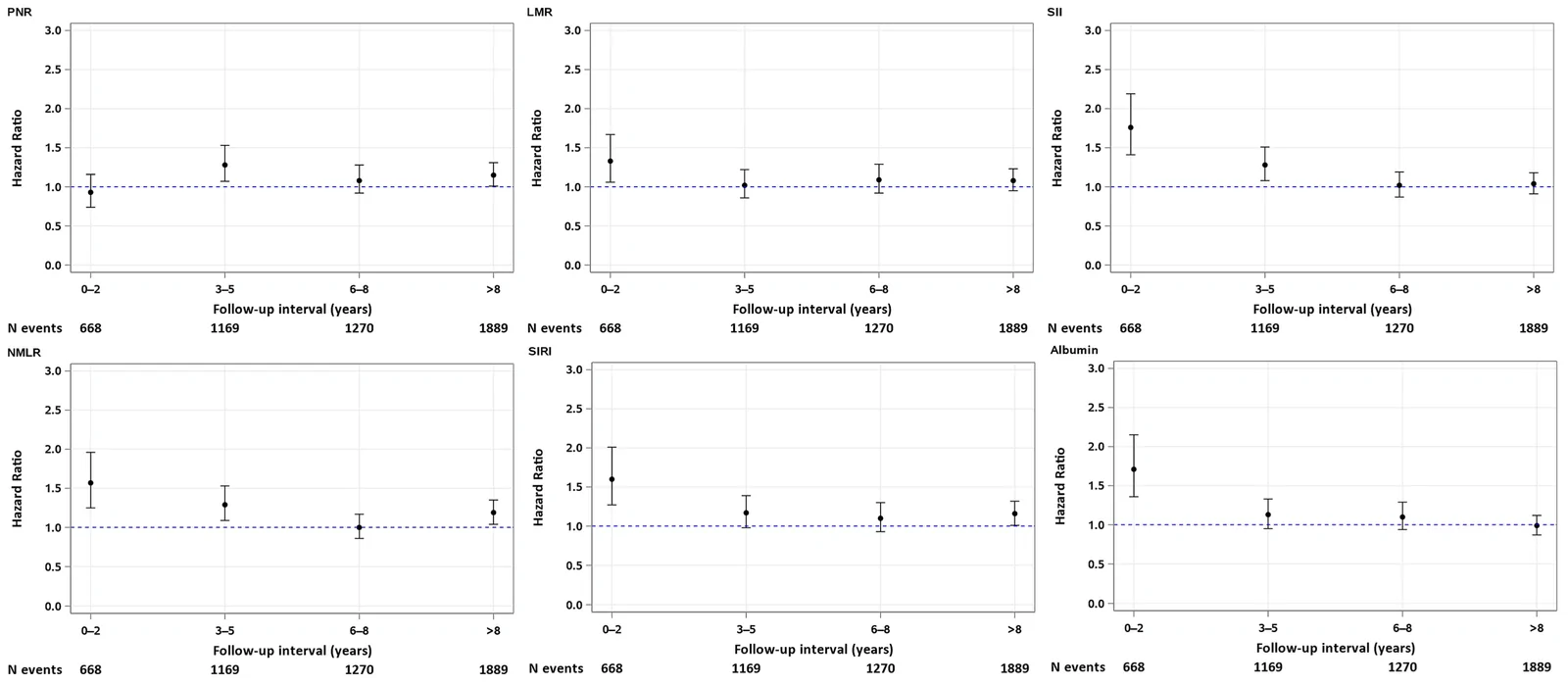
Hazard ratios (95% confidence intervals) for the associations between various biomarkers and colorectal cancer risk (highest vs. lowest quartile; for PNR, LMR, and albumin, the direction of comparison is reversed; lowest vs. highest quartile) during follow-up years 0–2, 3–5, 6–8, and beyond 8 years. Abbreviations: CRP: C-reactive protein; LMR: Lymphocyte-to-monocyte ratio; NMLR: Neutrophil-monocyte-to-lymphocyte ratio; NLR: Neutrophil-to-lymphocyte ratio; PLR: Platelet-to-lymphocyte ratio; PNR: Platelet-to-neutrophil ratio; SIRI: Systemic inflammation response index; SII: Systemic immune-inflammation index.

As displayed in Figure 2 and Figure 3 and Supplementary Tables S3 and S4, the association between baseline systemic inflammatory biomarkers and CRC risk was most pronounced during the early years of follow-up, particularly in the first year, where it was strongest. In contrast, the association was either absent or substantially weaker in later follow-up periods. This pattern was most evident for CRP, where elevated baseline levels were strongly associated with CRC risk within the first year (HR for highest vs. lowest quartile: 3.03; 95% CI: 2.06–4.45) and during the first two years (HR for highest vs. lowest quartile: 2.25; 95% CI: 1.75–2.89). However, the association weakened considerably and became non-significant during years 3–5 (HR for highest vs. lowest quartile: 1.13; 95% CI: 0.94–1.36), with virtually no association observed in the later follow-up years. Nevertheless, as shown in Figure 2, in a conventional cohort study analysis, without accounting for reverse causality, statistically significant overall associations between most of these biomarkers and CRC risk for up to more than 10 years of follow-up were found (for example, for CRP: HR for highest vs. lowest quartile 1.17; 95% CI: 1.07–1.28).

A similar pattern was observed for other systemic inflammatory biomarkers. For instance, an increase in SII levels was associated with a 1.76-fold increase in CRC risk for the highest vs. lowest quartile (95% CI: 1.41–2.19) during the first two years. The association was notably attenuated but remained statistically significant when the full follow-up period was considered (HR for highest vs. lowest quartile: 1.17; 95% CI: 1.08–1.26). However, no significant association was observed beyond five years of follow-up. An exception to this pattern was PNR, for which no such trend was evident. 

Restricted cubic spline analyses over the complete follow-up period showed linear associations between the natural log-transformed values of CRP, NLR, SII, and albumin and CRC risk, with no evidence of non-linearity (*p* for non-linearity = 0.9040, 0.4337, 0.7717, and 0.2041, respectively; Supplementary Figure S1).

**Figure 4:**
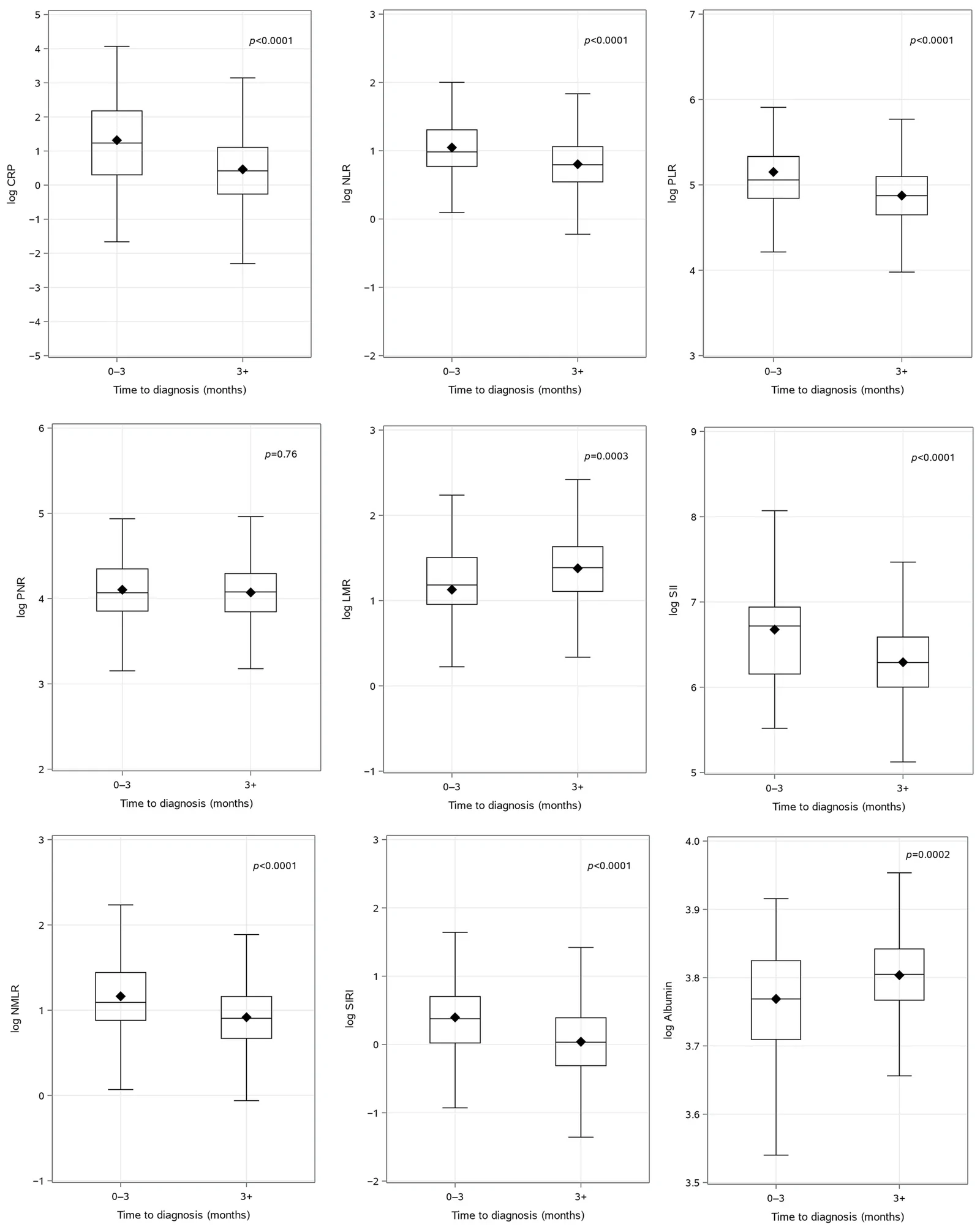
Distribution of log-transformed baseline inflammatory biomarkers among colorectal cancer cases diagnosed within 3 months (n = 62) and ≥3 months after baseline (n = 4934).

Among participants who developed CRC, the distribution of baseline inflammatory biomarkers varied according to proximity to CRC diagnosis (Figure 4). For all biomarkers except PNR, log-transformed biomarker levels differed between cases diagnosed within 0–3 months and those diagnosed ≥3 months after recruitment, whereas no clear difference was observed for PNR (*p* = 0.76). These findings are presented as descriptive comparisons of biomarker distributions according to proximity to CRC diagnosis. 

Results of the sensitivity analyses excluding participants with extreme biomarker values, as previously defined, are presented in Supplementary Tables S5 and S6. After these exclusions, 297,367 participants, including 3782 incident CRC cases, remained in the analysis. The findings were consistent with the primary analyses, indicating that the observed associations and temporal patterns were not primarily driven by extreme biomarker values.

Analyses restricted to participants with available CRP measurements included 430,871 participants, among whom 5568 developed CRC. The associations across CRP quartiles showed patterns comparable to those observed in the primary analyses using the common complete-biomarker population. Analyses using the natural logarithm of CRP similarly supported the temporal pattern of stronger associations during earlier follow-up, with attenuation over longer follow-up. Associations estimated per 1-SD increase in the natural logarithm of CRP followed the same pattern. Stronger associations were observed among men than women and for colon than rectal cancer; the *p* values for interaction by sex and for heterogeneity by cancer site, calculated over the complete follow-up period, were both 0.0004. Results of these additional analyses are presented in Supplementary Tables S7 and S8.

In a further sensitivity analysis within the CRP-specific complete-case population, excluding participants with prevalent IBD had essentially no effect on the associations between CRP and CRC risk. The association remained strongest during the first two years and attenuated over time, with no statistically significant associations observed during the later mutually exclusive follow-up intervals (Supplementary Table S9).

In the Fine–Gray competing-risk analysis, accounting for death before CRC diagnosis as a competing event, the associations between CRP and CRC risk were strongest during early follow-up and progressively attenuated across longer cumulative follow-up periods. These findings were consistent with the primary Cox regression analyses (Supplementary Table S10).

## 4. Discussion

Based on our findings, the association between levels of systemic inflammatory biomarkers and CRC risk that has been seen in many cohort studies may reflect a strong association shortly after recruitment. Even though the association diminishes with increasing length of follow-up, an apparent overall association may still persist after many follow-up years. This interpretation is further supported by the observed differences in baseline inflammatory biomarker distributions between CRC cases diagnosed very soon after recruitment (within 0–3 months) and those diagnosed later (≥3 months), suggesting that systemic inflammatory biomarker levels measured at baseline may partly reflect systemic responses associated with preclinical CRC.

The systemic inflammatory processes reflected by the biomarkers examined in our study may contribute to CRC carcinogenesis through several mechanisms. Interleukin-6 (IL-6), produced by immune, stromal, and tumor cells, regulates the hepatic acute-phase response, during which C-reactive protein concentrations increase and albumin concentrations decrease. IL-6 can also activate signal transducer and activator of transcription 3 (STAT3) signaling in CRC cells, promoting proliferation, angiogenesis, and tumor invasion [8,10,11]. Inflammatory cells may produce reactive oxygen and nitrogen species that cause DNA damage, while neutrophils and monocytes/macrophages may release mediators that support tumor progression; conversely, cytotoxic lymphocytes contribute to antitumor immunity [8,10,14]. Platelets may further promote angiogenesis, tumor growth, and invasion, and blood cell-derived ratios may therefore reflect the balance between tumor-promoting inflammation and antitumor immunity [11,14]. However, because inflammatory mediators may also originate from tumor and stromal cells within the tumor microenvironment, these biomarkers may reflect systemic responses to preclinical disease. The temporal attenuation observed in our analyses supports a contribution from this mechanism without excluding a potential causal role of systemic inflammation.

The temporal patterns observed in our study are consistent with findings from previous, smaller studies. For example, Song et al., in a prospective cohort of 51,529 US male health professionals, reported a positive association between certain systemic inflammatory biomarkers, including CRP, and CRC risk only during the first two years of follow-up. During that period, the multivariable relative risk (RR) for the highest versus the lowest quartile of log-transformed CRP was 4.73 (95% CI: 1.31–17.1). No association was observed beyond two years, with a multivariable RR of 0.90 (95% CI: 0.54–1.52) [21].

In a nested case-control study within the population-based Northern Sweden Health and Disease Study (NSHDS) cohorts, the association between one unit increase in ln plasma CRP concentrations and CRC risk was statistically significant, only for advanced tumors and blood samples taken less than 5 years before diagnosis, with an odds ratio (95% CI) of 1.32 (1.01–1.73) [22].

Other systemic inflammatory biomarkers examined in our study have previously been mostly investigated as diagnostic or prognostic tools for CRC. In one study assessing the potential role of NLR, PLR, and SII in early CRC diagnosis, all biomarkers showed higher values when measured closer to diagnosis, in individuals with more advanced CRC stages, and in comparison to healthy controls [6]. Additionally, elevated levels of these biomarkers—above the cut-off points defined in the study—were associated with an increased risk of CRC, consistent with our findings. The odds ratios (95% CIs) were 6.47 (4.17–10.04) for NLR ≥ 2.28 vs. <2.28, 6.53 (4.16–10.26) for PLR ≥ 122.40 vs. <122.40, and 9.88 (5.80–16.84) for SII ≥ 616.50 vs. <616.50. The biomarkers were calculated using blood tests conducted both prior to diagnosis (median: 6 months; interquartile range: 4–8 months) and at the time of diagnosis. However, the associations were not assessed at different time points before diagnosis.

Another recent study using UK Biobank data evaluated the association between inflammatory bowel disease (IBD)—a chronic condition characterized by chronic inflammation of the gastrointestinal tract—and CRC, and found a significant positive association with CRC risk even after accounting for reverse causality [23]. In this study the mediating role of various inflammatory biomarkers—including albumin, CRP, hemoglobin, platelets, neutrophils, lymphocytes, PLR, NLR, and SII—in the association between IBD and CRC was also investigated. While CRP and NLR were identified as significant mediators in the progression from IBD to CRC, reverse causality was not accounted for in this analysis.

Regarding a possible role of reverse causality, the length of preclinical sojourn time, i.e., the time period during which cancer is already prevalent but yet undiagnosed, is of particular interest. For CRC, mean sojourn time has been estimated in the order of two to six years [24,25]. Preclinical sojourn time in more advanced stage, which might be the most relevant period with respect to increases in systemic inflammatory biomarkers, would be expected to be somewhat lower. Overall, these temporal patterns observed in our analysis would seem consistent with the hypothesized potential role of reverse causality. This suggestion is further supported by a recent Mendelian randomization study which did not find an association between genetically determined CRP and CRC risk [26]. Nevertheless, although the stronger associations close to baseline and their attenuation in the mutually exclusive later follow-up intervals support a potential contribution of preclinical disease, time-stratified observational analyses cannot exclude a causal contribution of systemic inflammation to CRC development, and both mechanisms may coexist.

A UK Biobank analysis examined the longitudinal associations of four blood cell ratios (SII, NLR, PLR, LMR) with the risk of several cancers, including CRC, and their potential utility as early-detection biomarkers, using flexible parametric survival models [27]. The study provides evidence that hazard ratios are higher close to diagnosis, supporting the idea that systemic inflammation may reflect preclinical disease and the potential of these biomarkers for earlier detection of cancer. Our approach extends these findings by focusing specifically on CRC, examining a broader range of circulating inflammatory biomarkers, and using interval-specific analyses to assess whether associations persist after excluding increasingly early periods of follow-up. We found that many overall associations between inflammatory biomarkers and CRC risk are substantially attenuated or become statistically insignificant when early follow-up periods are excluded, providing further evidence that the temporal pattern of these associations is important for their interpretation. 

PNR was the only biomarker that did not show the temporal pattern observed for the other biomarkers. One possible explanation is its distinct cellular composition. PNR is derived from platelet and neutrophil counts and, unlike all the other cell-derived inflammatory indices examined, does not incorporate the circulating lymphocyte count. Systemic immune dysregulation in CRC involves alterations in both myeloid and lymphoid cell populations, while platelets also participate in cancer-associated inflammatory and immune processes [14,28,29]. Because platelet and neutrophil counts may change concurrently, their ratio may remain relatively stable, whereas lymphocyte-containing indices may capture differential changes between systemic inflammatory responses and lymphocyte-mediated immunity. This compositional difference may partly explain why PNR did not show stronger associations close to baseline followed by attenuation during subsequent follow-up.

It is important to point out that our analysis focused on biomarkers of systemic inflammation rather than local inflammation in the large bowel. It is well-established that such on-site inflammation, e.g. among people suffering from IBD, goes along with increased CRC risk and requires dedicated therapeutic and preventive action [30,31].

It is also worth noting that anti-inflammatory chemoprevention of CRC has been proposed and investigated beyond the specific high-risk groups, such as people with IBD; however, the findings of existing studies have been controversial and considered insufficient. The most commonly investigated agent is aspirin, which has shown inconsistent results in various randomized clinical trials, and the evidence regarding its role in reducing CRC incidence and mortality remains unclear [32]. Non-aspirin NSAIDs have also been explored for CRC prevention [33]. Although some trials have shown promising effects in preventing colorectal adenomas, their use is associated with increased risks of serious side effects, including cardiovascular and upper gastrointestinal events. The present findings provide additional context for interpreting the role of systemic inflammation in CRC risk and underscore the complexity of this relationship, which may be relevant when considering systemic inflammation as a target for CRC chemoprevention.

### Strengths and Limitations

Our study offers several strengths. First, biomarker and blood cell count measurements were obtained using standardized methods and were available for the entire cohort, allowing analyses restricted to shorter follow-up periods. Second, the extensive data collected by the UK Biobank enabled adjustment for a wide range of potential confounding variables in the Cox regression models. However, there are important limitations to note. Despite adjustment for a wide range of potential confounders, residual confounding due to unmeasured or imperfectly measured factors cannot be excluded, and covariates measured only at baseline did not account for changes over time. Biomarker and cell count measurements were only taken at a single time point at baseline, which may introduce bias in assessing their association with CRC risk. Furthermore, acute infections around the time of biomarker assessment may have caused transient elevations in inflammatory biomarkers, which could have affected the classification of participants according to their baseline biomarker levels. Other comorbidities associated with systemic inflammation and the use of corticosteroids or immunosuppressive medications were not comprehensively accounted for and may have influenced baseline biomarker levels or contributed to residual confounding. Moreover, evidence indicates that the UK Biobank cohort is healthier and less socioeconomically deprived than the general UK population (“healthy volunteer” selection bias) [34]. Additionally, information on cancer stage is not yet available in the UK Biobank dataset, which prevented us from conducting detailed analyses stratified by CRC stage at diagnosis. This represents an important limitation when interpreting the temporal patterns observed in our study, as we could not determine whether the stronger associations close to CRC diagnosis reflect occult advanced disease, earlier-stage disease, or differences in the timing of diagnosis. Finally, since the majority of participants are of White European descent, the findings may not be applicable to other ethnic groups.

## 5. Conclusions

Our findings suggest that the observed associations between systemic inflammatory biomarkers and CRC risk may partly reflect reverse causality, with systemic inflammatory responses arising from preclinical disease, rather than solely a causal role of systemic inflammation in CRC development. This potential link to preclinical disease warrants further investigation of these biomarkers for risk stratification to support early CRC detection.

## Figures and Tables

**Table 1:** Baseline characteristics of the cohort population.

Characteristic	Whole Cohort (N = 383,850)	CRC Cases (n = 4996)
Age at baseline, years	57 (50–63)	62 (56–66)
Female, n (%)	202,021 (52.6)	2124 (42.5)
Non-white, n (%)	20,830 (5.5)	152 (3.1)
Country of recruitment		
England	339,130 (88.4)	4383 (87.7)
Scotland	27,988 (7.3)	464 (9.3)
Wales	16,732 (4.4)	149 (3.0)
Townsend deprivation index	−2.1 (−3.6–0.5)	−2.3 (−3.7–0.4)
Educational qualifications, n (%)		
Higher academic/professional	187,624 (49.5)	2266 (46.1)
Lower academic/vocational	127,541 (33.6)	1649 (33.5)
None	64,123 (16.9)	1005 (20.4)
CRP, mg/L	1.3 (0.7–2.7)	1.5 (0.8–3.1)
NLR	2.1 (1.7–2.8)	2.2 (1.7–2.9)
PLR	132.0 (105.3–165.8)	131.1 (104.6–164.4)
PNR	61.5 (48.8–77.3)	59.0 (46.8–73.3)
LMR	4.2 (3.3–5.3)	4.0 (3.0–5.1)
SII	527.4 (391.7–713.1)	540.7 (404.6–730.7)
NMLR	2.4 (1.9–3.0)	2.5 (2.0–3.2)
SIRI	1.0 (0.7–1.4)	1.0 (0.7–1.5)
Albumin, g/L	45.2 (43.5–46.9)	44.9 (43.2–46.6)
BMI, kg/m^2^	26.8 (24.2–29.9)	27.3 (24.8–30.3)
Pack-years of smoking, years	0.0 (0.0–11.1)	0.0 (0.0–20.0)
Alcohol consumption, n (%)		
Never	30,421 (7.9)	362 (7.3)
Special occasion only	43,310 (11.3)	526 (10.6)
1–3 times a month	42,521 (11.1)	469 (9.4)
Once or twice a week	99,075 (25.9)	1163 (23.3)
3–4 times a week	89,288 (23.3)	1162 (23.3)
Daily or almost daily	78,371 (20.5)	1301 (26.1)
Physical activity (IPAQ groups), n (%)		
Low	54,893 (18.4)	718 (18.5)
Moderate	120,587 (40.5)	1616 (41.7)
High	122,253 (41.1)	1538 (39.7)
Fruit intake, pieces/day	2.5 (1.0–4.0)	2.0 (1.0–4.0)
Vegetable intake, tablespoons/day	4.0 (3.0–6.0)	4.0 (3.0–6.0)
Whole grain intake, servings/week	7.0 (0.0–14.0)	7.0 (0.0–14.0)
Refined grain intake, servings/week	6.0 (0.0–12.0)	7.0 (0.0–14.0)
Fish intake <2 times a week, n (%)	184,813 (48.3)	2290 (46.0)
Red meat intake, n (%)		
Never	25,495 (6.7)	233 (4.7)
Less than once a week	142,394 (37.5)	1772 (35.9)
Once a week	82,344 (21.7)	1075 (21.8)
≥2 times a week	129,301 (34.1)	1859 (37.6)
Processed meat intake, n (%)		
Never	35,103 (9.2)	327 (6.6)
Less than once a week	115,342 (30.2)	1405 (28.2)
Once a week	111,557 (29.2)	1510 (30.3)
≥2 times a week	120,442 (31.5)	1742 (35.0)
History of CRC screening, n (%)	114,655 (30.4)	1768 (35.9)
Family history of CRC, n (%)	41,388 (11.0)	761 (15.5)
Regular use of NSAIDs/aspirin, n (%)	117,559 (30.6)	1585 (31.7)

The numbers show median (interquartile range), unless otherwise specified. Values are based on observed data before multiple imputation. Number of missing values for each covariate in the total cohort: age (0), sex (0), ethnicity (1824), Country of recruitment (0), Townsend deprivation index (481), educational qualifications (4562), BMI (1509), pack-years of smoking (58,647), alcohol consumption (864), physical activity (86,117), fruit intake (1376), vegetable intake (3179), whole grain intake (4880), refined grain intake (5544), fish intake (1289), read meat intake (4316), processed meat intake (1406), history of CRC screening (7127), family history of CRC (7866), NSAID use (83). Abbreviations: BMI body mass index; CRC colorectal cancer; CRP C-reactive protein; IPAQ international physical activity questionnaire; LMR lymphocyte-to-monocyte ratio; NLR neutrophil-to-lymphocyte ratio; NMLR neutrophil-monocyte-to-lymphocyte ratio; NSAIDs non-steroidal anti-inflammatory drugs; PLR platelet-to-lymphocyte ratio; PNR platelet-to-neutrophil ratio; SII systemic immune-inflammation index; SIRI systemic inflammation response index.

## Data Availability

Data was re-used with the permission of the UK Biobank. The UK Biobank is an open-access resource and bona fide researchers can apply to use the UK Biobank dataset by registering and applying at https://www.ukbiobank.ac.uk/use-our-data/apply-for-access/ (accessed on 27 September 2026). The data and analysis codes used for this study are going to be available on the UK Biobank website for registered researchers at the UK Biobank and an application fee.

## Supplementary Materials

The following supporting information can be downloaded at: https://www.mdpi.com/article/10.3390/cancers18193190/s1, Table S1. Frequency of missing values for each variable. Table S2. Characteristics of participants included in the analytical cohort with complete biomarker data and participants excluded due to at least one missing biomarker. Table S3. Hazard ratios (95% confidence intervals) of colorectal cancer risk across quartiles of various biomarkers, for cumulative follow-up periods. Table S4. Hazard ratios (95% confidence intervals) of colorectal cancer risk across quartiles of various biomarkers, during follow-up years 0–2, 3–5, 6–8, and beyond 8 years. Table S5. Hazard ratios (95% confidence intervals) of colorectal cancer risk across quartiles of various biomarkers, for cumulative follow-up periods, after excluding extreme biomarker values. Table S6. Hazard ratios (95% confidence intervals) of colorectal cancer risk across quartiles of various biomarkers, during follow-up years 0–2, 3–5, 6–8, and beyond 8 years, after excluding extreme biomarker values. Table S7. Associations between baseline C-reactive protein and colorectal cancer risk, for cumulative follow-up periods, according to sex and cancer site, in the CRP-specific complete-case population. Table S8. Associations between baseline C-reactive protein and colorectal cancer risk, during follow-up years 0–2, 3–5, 6–8, and beyond 8 years, according to sex and cancer site, in the CRP-specific complete-case population. Table S9. Associations between baseline C-reactive protein and colorectal cancer risk, during various follow-up periods, in the CRP-specific complete-case population, after excluding participants with prevalent inflammatory bowel disease (IBD). Table S10. Fine–Gray competing-risk analysis of the associations between C-reactive protein and colorectal cancer risk across cumulative follow-up periods in the CRP-specific complete-case population. Figure S1. Restricted cubic spline analyses of the associations between inflammatory biomarkers and colorectal cancer risk.

## Abbreviations

The following abbreviations are used in this manuscript:

BMI	body mass index
CRC	colorectal cancer
CI	confidence interval
CRP	C-reactive protein
HR	hazard ratio
ICD	international statistical classification
IPAQ	international physical activity questionnaire
IQR	interquartile range
LMR	lymphocyte-to-monocyte ratio
LYMPH	lymphocyte
MONO	monocyte
NEUT	neutrophil
NLR	neutrophil-to-lymphocyte ratio
NMLR	neutrophil-monocyte-to-lymphocyte ratio
NSAIDs	non-steroidal anti-inflammatory drugs
PLR	platelet-to-lymphocyte ratio
PLT	platelet
PNR	platelet-to-neutrophil ratio
SII	systemic immune-inflammation index
SIRI	systemic inflammation response index
