# Supplementary material for: Systemic Inflammation and Colorectal Cancer: Assessing the Role of Reverse Causality
Source: Cancers (Basel). 2026 Oct 2;18(19):3190. doi: 10.3390/cancers18193190 (PMC13663995; doi:10.3390/cancers18193190)
Supplement: Supplementary file 1 [file cancers-18-03190-s001.zip › cancers-4475283-supplementary.pdf]

Supplementary material

Systemic inflammation and colorectal cancer: chicken or egg?

Table S1. Frequency of Missing Values for each variable.

Table S2. Characteristics of participants included in the analytical cohort with complete biomarker data and participants excluded due to at least one missing biomarker.

Table S3. Hazard ratios (95% confidence intervals) of colorectal cancer risk across quartiles of various biomarkers, for cumulative follow-up periods.

Table S4. Hazard ratios (95% confidence intervals) of colorectal cancer risk across quartiles of various biomarkers, during follow-up years 0–2, 3–5, 6–8, and beyond 8 years.

Table S5. Hazard ratios (95% confidence intervals) of colorectal cancer risk across quartiles of various biomarkers, for cumulative follow-up periods, after excluding extreme biomarker values.

Table S6. Hazard ratios (95% confidence intervals) of colorectal cancer risk across quartiles of various biomarkers, during follow-up years 0–2, 3–5, 6–8, and beyond 8 years, after excluding extreme biomarker values.

Table S7. Associations between baseline C-reactive protein and colorectal cancer risk, for cumulative follow-up periods, according to, sex and cancer site, in the CRP-specific complete-case population.

Table S8. Associations between baseline C-reactive protein and colorectal cancer risk, during follow-up years 0–2, 3–5, 6–8, and beyond 8 years, according to, sex and cancer site, in the CRP-specific complete-case population.

Table S9. Associations between baseline C-reactive protein and colorectal cancer risk, during various follow-up periods, in the CRP-specific complete-case population, after excluding participants with prevalent inflammatory bowel disease (IBD).

Table S10. Fine–Gray competing-risk analysis of the associations between C-reactive protein and colorectal cancer risk across cumulative follow-up periods in the CRP-specific complete-case population.

Figure S1. Restricted cubic spline analyses of the associations between inflammatory biomarkers and colorectal cancer risk.

Table S1. Frequency of Missing Values for each variable.

**Variable**	**n (%) of missing data**
Age	0 (0.0)
Sex	0 (0.0)
Ethnic background	1,824 (0.5)
Townsend deprivation index	481 (0.1)
Educational qualifications	4,562 (1.2)
Body mass index	1,509 (0.4)
Pack-years of smoking	58,647 (15.3)
Alcohol intake frequency	864 (0.2)
Physical activity	86,117 (22.4)
Fruit intake	1,376 (0.4)
Vegetable intake	3,179 (0.8)
Whole grain intake	4,880 (1.3)
Refined grain intake	5,544 (1.4)
Fish intake	1,289 (0.3)
Red meat intake	4,316 (1.1)
Processed meat intake	1,406 (0.4)
History of bowel cancer screening	7,127 (1.9)
Family history of colorectal cancer	7,866 (2.0)
Use of non-steroidal anti-inflammatory drugs (NSAIDs)	83 (0.0)

Table S2. Characteristics of participants included in the analytical cohort with complete biomarker data and participants excluded due to at least one missing biomarker.

Characteristic	Complete biomarkers, n (%)	≥1 biomarker missing, n (%)	P-value
**Sociodemographic characteristics**			
Age, median (IQR)	57 (50–63)	57 (50–63)	
Female	202021 (52.6)	43691 (56.3)	<.0001
Non-White	20830 (5.5)	4936 (6.4)	<.0001
Townsend deprivation index, median (IQR)	−2.1 (−3.6–0.5)	−2.1 (−3.6–0.8)	
Recruitment country			<.0001
England	339130 (88.4)	70391 (90.7)	
Scotland	27988 (7.3)	4882 (6.3)	
Wales	16732 (4.4)	2379 (3.1)	
**Lifestyle characteristics**			
Smoking status			0.0004
Never	210291 (55.1)	42801 (55.7)	
Former	131136 (34.3)	25820 (33.6)	
Current	40534 (10.6)	8250 (10.7)	
Alcohol consumption			<.0001
Never	30421 (7.9)	6560 (8.5)	
Special occasion only	43310 (11.3)	9221 (12.0)	
1-3 times a month	42521 (11.1)	8709 (11.3)	
Once or twice a week	99075 (25.9)	19830 (25.7)	
3-4 times a week	89288 (23.3)	17559 (22.8)	
Daily or almost daily	78371 (20.5)	15230 (19.8)	
**Clinical characteristics**			
Colorectal cancer incidence	4996 (1.3)	983 (1.3)	0.42
**Sample collection and laboratory processing characteristics**			
Acquisition year			
Lymph/ Neut/Mono			<.0001
2007	38813 (10.1)	5304 (6.8)	
2008	146988 (38.3)	17588 (22.7)	
2009	129483 (33.7)	23025 (29.7)	
2010	68566 (17.9)	9132 (11.8)	
Missing	0 (0.0)	22603 (29.1)	
Platelet			<.0001
2007	38813 (10.1)	53.4 (6.8)	
2008	146988 (38.3)	17591 (22.7)	
2009	129483 (33.7)	23835 (30.7)	
2010	68566 (17.9)	9131 (11.8)	
Missing	0 (0.0)	21791 (28.1)	
Assay year			
Albumin			<.0001
2015	5228 (1.4)	4512 (5.8)	
2016	141658 (36.9)	25424 (32.7)	
2017	236964 (61.7)	21694 (27.9)	
Missing	0 (0.0)	26022 (33.5)	
C-reactive protein			<.0001
2015	5228 (1.4)	4302 (5.5)	
2016	141658 (36.9)	25360 (32.7)	
2017	236964 (61.7)	21622 (27.8)	
Missing	0 (0.0)	26368 (34.0)	

Table S3. Hazard ratios (95% confidence intervals) of colorectal cancer risk across quartiles of various biomarkers, for cumulative follow-up periods.

Biomarker/follow-up years included	0–2 (N=383,850)			0–5 (N=383,850)			0–8 (N=383,850)			Complete follow-up (N=383,850)		
**CRP**	Person-years	n Cases	HR (95% CI)	Person-years	n Cases	HR (95% CI)	Person-years	n Cases	HR (95% CI)	Person-years	n Cases	HR (95% CI)
Q1	189,232	100	Ref.	471,443	327	Ref.	749,763	596	Ref.	1,100,136	975	Ref.
Q2	193,233	140	1.22 (0.94-1.58)	480,921	417	1.07 (0.92-1.23)	763,796	737	1.03 (0.93-1.15)	1,116,745	1,222	1.05 (0.97-1.15)
Q3	192,105	176	1.50 (1.16-1.93)	477,519	498	1.21 (1.05-1.40)	757,317	862	1.14 (1.02-1.27)	1,104,687	1,357	1.10 (1.01-1.20)
Q4	191,077	252	2.25 (1.75-2.89)	473,556	595	1.46 (1.25-1.69)	748,573	912	1.21 (1.08-1.36)	1,086,961	1,442	1.17 (1.07-1.28)
**NLR**												
Q1	191,542	129	Ref.	476,808	371	Ref.	757,522	672	Ref.	1,105,713	1,094	Ref.
Q2	191,400	146	1.07 (0.84-1.35)	476,358	427	1.09 (0.95-1.26)	756,521	741	1.05 (0.94-1.16)	1,106,843	1,209	1.05 (0.97-1.14)
Q3	191,560	166	1.17 (0.93-1.48)	476,304	468	1.16 (1.02-1.34)	755,660	786	1.08 (0.98-1.20)	1,104,309	1,249	1.06 (0.98-1.15)
Q4	191,144	227	1.49 (1.19-1.85)	473,970	571	1.34 (1.17-1.53)	749,747	908	1.18 (1.07-1.31)	1,091,665	1,444	1.18 (1.09-1.28)
**PLR**												
Q1	191,425	148	Ref.	475,599	454	Ref.	753,495	798	Ref.	1,083,905	1,295	Ref.
Q2	191,475	152	1.11 (0.89-1.40)	476,229	449	1.07 (0.94-1.22)	755,619	763	1.03 (0.93-1.14)	1,100,109	1,251	1.02 (0.94-1.10)
Q3	191,436	167	1.27 (1.01-1.59)	476,209	467	1.16 (1.01-1.32)	756,212	774	1.08 (0.98-1.20)	1,109,849	1,234	1.03 (0.95-1.12)
Q4	191,310	201	1.55 (1.25-1.92)	475,403	467	1.18 (1.04-1.35)	754,125	772	1.11 (1.00-1.22)	1,114,666	1,216	1.03 (0.95-1.12)
**PNR**												
Q4	191,551	146	Ref.	477,120	356	Ref.	758,836	610	Ref.	1,124,236	1,034	Ref.
Q3	191,483	143	0.87 (0.69-1.10)	476,490	436	1.10 (0.96-1.27)	756,883	760	1.12 (1.01-1.25)	1,110,158	1,203	1.07 (0.99-1.16)
Q2	191,423	190	1.05 (0.85-1.31)	475,948	500	1.16 (1.01-1.33)	755,081	830	1.13 (1.02-1.26)	1,098,696	1,311	1.10 (1.01-1.19)
Q1	191,189	189	0.93 (0.74-1.16)	473,881	545	1.13 (0.99-1.30)	748,649	907	1.11 (1.00-1.24)	1,075,441	1,448	1.13 (1.04-1.22)
**LMR**												
Q4	197,100	129	Ref.	490,645	383	Ref.	779,524	648	Ref.	1,139,635	1,082	Ref.
Q3	186,056	140	1.05 (0.82-1.33)	463,067	409	1.04 (0.90-1.19)	735,402	706	1.06 (0.95-1.18)	1,075,390	1,137	1.03 (0.95-1.12)
Q2	194,356	163	1.06 (0.83-1.34)	483,189	467	1.03 (0.90-1.19)	766,547	795	1.04 (0.93-1.15)	1,119,328	1,262	1.01 (0.93-1.10)
Q1	188,133	236	1.33 (1.06-1.67)	466,539	578	1.13 (0.99-1.30)	737,976	958	1.11 (1.00-1.24)	1,074,176	1,515	1.10 (1.01-1.20)
**SII**												
Q1	191,540	126	Ref.	476,578	382	Ref.	756,506	699	Ref.	1,098,311	1,143	Ref.
Q2	191,471	142	1.13 (0.89-1.44)	476,487	439	1.15 (1.01-1.32)	756,508	765	1.10 (0.99-1.22)	1,104,712	1,244	1.08 (1.00-1.17)
Q3	191,461	176	1.40 (1.12 (1.77)	476,087	466	1.23 (1.07-1.41)	755,574	775	1.12 (1.01-1.24)	1,106,202	1,271	1.11 (1.02-1.20)
Q4	191,173	224	1.76 (1.41-2.19)	474,287	550	1.44 (1.26-1.64)	750,861	868	1.25 (1.13-1.38)	1,099,305	1,338	1.17 (1.08-1.26)
**NMLR**												
Q1	191,562	121	Ref.	476,948	353	Ref.	757,840	650	Ref.	1,106,762	1,074	Ref.
Q2	191,154	149	1.16 (0.91-1.47)	475,740	431	1.16 (1.01-1.33)	755,641	739	1.08 (0.97-1.20)	1,105,349	1,187	1.05 (0.97-1.14)
Q3	191,890	168	1.25 (0.99-1.58)	477,138	477	1.23 (1.07-1.41)	756,911	796	1.12 (1.01-1.24)	1,105,835	1,260	1.08 (1.00-1.17)
Q4	191,040	230	1.57 (1.25-1.96)	473,613	576	1.39 (1.21-1.59)	749,058	922	1.21 (1.09-1.34)	1,090,584	1,475	1.20 (1.11-1.30)
**SIRI**												

Biomarker/follow-up years included	0–2 (N=383,850)			0–5 (N=383,850)			0–8 (N=383,850)			Complete follow-up (N=383,850)		
Q1	191,200	116	Ref.	476,360	337	Ref.	757,549	586	Ref.	1,111,731	995	Ref.
Q2	191,849	139	1.07 (0.84-1.38)	477,476	409	1.09 (0.94-1.26)	758,385	723	1.11 (0.99-1.24)	1,110,410	1,157	1.06 (0.97-1.15)
Q3	191,531	154	1.09 (0.86-1.40)	476,280	478	1.17 (1.02-1.35)	755,674	812	1.15 (1.03-1.28)	1,102,788	1,287	1.10 (1.01-1.19)
Q4	191,067	259	1.60 (1.27-2.01)	473,324	613	1.32 (1.15-1.52)	747,842	986	1.23 (1.10-1.36)	1,083,600	1,557	1.20 (1.10-1.30)
**Albumin**												
Q4	192,426	116	Ref.	478,995	359	Ref.	760,981	639	Ref.	1,107,732	1,079	Ref.
Q3	191,838	149	1.19 (0.93-1.51)	477,324	424	1.08 (0.94-1.25)	757,862	729	1.05 (0.95-1.17)	1,107,374	1,176	1.00 (0.92-1.09)
Q2	190,644	166	1.26 (0.99-1.60)	474,013	481	1.17 (1.02-1.34)	751,876	811	1.12 (1.01-1.25)	1,099,846	1,286	1.05 (0.97-1.14)
Q1	190,738	237	1.71 (1.36-2.15)	473,108	573	1.32 (1.15-1.50)	748,732	928	1.22 (1.10-1.35)	1,093,579	1,455	1.13 (1.04-1.22)

Models are adjusted for age, sex, BMI, ethnicity, Townsend deprivation index, educational qualifications, physical activity, alcohol consumption, packyears of smoking, intake of vegetables, fruit, fish, whole and refined grains, and red and processed meat, history of CRC screening, family history of CRC, and regular intake of NSAIDs. The cut-offs for the biomarkers were as follows: CRP (0.65, 1.32, 2.74); NLR (1.66, 2.13, 2.76); PLR (105.27, 132.00, 165.83); PNR (48.82, 61.51, 77.33); LMR (3.25, 4.20, 5.33); SII (391.67, 527.40, 713.09); NMLR (1.88, 2.38, 3.05); SIRI (0.68, 0.95, 1.35); and albumin (43.52, 45.22, 46.94). For PNR, LMR, and albumin, the direction of comparison is reversed, and Q4 was used as the reference category; for all other biomarkers, Q1 was used as the reference category.

Table S4. Hazard ratios (95% confidence intervals) of colorectal cancer risk across quartiles of various biomarkers, during follow-up years 0–2, 3–5, 6–8, and beyond 8 years.

Biomarker/follow-up years included	0–2 (N=383,850)			3–5 (N=381,720)			6–8 (N=376,388)			>8 (N=367,278)		
**CRP**	Person-years	n Cases	HR (95% CI)	Person-years	n Cases	HR (95% CI)	Person-years	n Cases	HR (95% CI)	Person-years	n Cases	HR (95% CI)
Q1	189,232	100	Ref.	471,107	227	Ref.	746,015	269	Ref.	1,085,970	379	Ref.
Q2	193,233	140	1.22 (0.94-1.58)	480,436	277	1.00 (0.83-1.19)	759,364	320	0.99 (0.84-1.17)	1,098,777	485	1.09 (0.95-1.24)
Q3	192,105	176	1.50 (1.16-1.93)	476,915	322	1.08 (0.90-1.29)	751,827	364	1.06 (0.90-1.25)	1,083,292	495	1.04 (0.91-1.20)
Q4	191,077	252	2.25 (1.75-2.89)	472,557	343	1.13 (0.94-1.36)	740,644	317	0.91 (0.76-1.09)	1,059,252	530	1.10 (0.95-1.27)
**NLR**												
Q1	191,542	129	Ref.	476,356	242	Ref.	753,201	301	Ref.	1,089,064	422	Ref.
Q2	191,400	146	1.07 (0.84-1.35)	475,908	281	1.11 (0.93-1.32)	751,954	314	0.99 (0.85-1.16)	1,089,111	468	1.06 (0.93-1.20)
Q3	191,560	166	1.17 (0.93-1.48)	475,719	302	1.16 (0.98-1.38)	750,326	318	0.98 (0.84-1.15)	1,084,053	463	1.03 (0.91-1.18)
Q4	191,144	227	1.49 (1.19-1.85)	473,033	344	1.25 (1.06-1.48)	742,370	337	0.99 (0.84-1.16)	1,065,062	536	1.17 (1.03-1.33)
**PLR**												
Q1	191,425	148	Ref.	474,958	306	Ref.	747,300	344	Ref.	1,060,823	497	Ref.
Q2	191,475	152	1.11 (0.89-1.40)	475,711	297	1.05 (0.89-1.23)	750,477	314	0.98 (0.84-1.14)	1,080,083	488	1.00 (0.88-1.13)
Q3	191,436	167	1.27 (1.01-1.59)	475,660	300	1.10 (0.94-1.30)	751,490	307	0.99 (0.85-1.16)	1,091,854	460	0.95 (0.83-1.07)
Q4	191,310	201	1.55 (1.25-1.92)	474,687	266	1.00 (0.85-1.19)	748,583	305	1.02 (0.87-1.19)	1,094,531	444	0.91 (0.80-1.04)
**PNR**												
Q4	191,551	146	Ref.	476,707	210	Ref.	755,124	254	Ref.	1,110,062	424	Ref.
Q3	191,483	143	0.87 (0.69-1.10)	475,984	293	1.26 (1.05-1.51)	752,360	324	1.15 (0.98-1.36)	1,092,994	443	1.00 (0.87-1.14)
Q2	191,423	190	1.05 (0.85-1.31)	475,348	310	1.23 (1.03-1.47)	749,692	330	1.09 (0.92-1.28)	1,078,424	481	1.05 (0.92-1.20)
Q1	191,189	189	0.93 (0.74-1.16)	472,976	356	1.28 (1.07-1.53)	740,675	362	1.08 (0.92-1.28)	1,045,811	541	1.15 (1.01-1.31)
**LMR**												
Q4	197,100	129	Ref.	490,184	254	Ref.	774,998	265	Ref.	1,122,761	434	Ref.
Q3	186,056	140	1.05 (0.82-1.33)	462,592	269	1.03 (0.87-1.23)	731,066	297	1.09 (0.92-1.28)	1,057,868	431	0.99 (0.86-1.13)
Q2	194,356	163	1.06 (0.83-1.34)	482,605	304	1.02 (0.86-1.21)	761,017	328	1.05 (0.89-1.24)	1,098,995	467	0.97 (0.85-1.11)
Q1	188,133	236	1.33 (1.06-1.67)	465,636	342	1.02 (0.86-1.22)	730,770	380	1.09 (0.92-1.29)	1,047,667	557	1.08 (0.95-1.23)
**SII**												
Q1	191,540	126	Ref.	476,096	256	Ref.	751,783	317	Ref.	1,079,458	444	Ref.
Q2	191,471	142	1.13 (0.89-1.44)	476,019	297	1.16 (0.99-1.38)	751,730	326	1.03 (0.89-1.21)	1,086,105	479	1.05 (0.93-1.20)
Q3	191,461	176	1.40 (1.12 (1.77)	475,498	290	1.14 (0.96-1.35)	750,373	309	0.98 (0.84-1.15)	1,087,028	496	1.08 (0.95-1.23)
Q4	191,173	224	1.76 (1.41-2.19)	473,402	326	1.28 (1.08-1.51)	743,964	318	1.02 (0.87-1.19)	1,074,700	470	1.04 (0.91-1.18)
**NMLR**												
Q1	191,562	121	Ref.	476,520	232	Ref.	753,604	297	Ref.	1,090,372	424	Ref.
Q2	191,154	149	1.16 (0.91-1.47)	475,277	282	1.16 (0.97-1.38)	751,146	308	0.98 (0.84-1.15)	1,087,881	448	1.01 (0.88-1.15)
Q3	191,890	168	1.25 (0.99-1.58)	476,559	309	1.22 (1.03-1.45)	751,593	319	0.98 (0.84-1.15)	1,085,328	464	1.02 (0.90-1.17)
Q4	191,040	230	1.57 (1.25-1.96)	472,659	346	1.29 (1.09-1.53)	741,508	346	1.00 (0.86-1.17)	1,063,710	553	1.19 (1.04-1.35)

**Biomarker/follow-up years included**	**0–2 (N=383,850)**			**3–5 (N=381,720)**			**6–8 (N=376,388)**			**>8 (N=367,278)**		
**SIRI**												
Q1	191,200	116	Ref.	476,007	221	Ref.	753,864	249	Ref.	1,097,296	409	Ref.
Q2	191,849	139	1.07 (0.84-1.38)	477,009	270	1.10 (0.92-1.32)	753,928	314	1.13 (0.96-1.34)	1,093,079	434	0.98 (0.86-1.13)
Q3	191,531	154	1.09 (0.86-1.40)	475,701	324	1.22 (1.02-1.45)	750,438	334	1.11 (0.94-1.31)	1,082,434	475	1.02 (0.90-1.17)
Q4	191,067	259	1.60 (1.27-2.01)	472,298	354	1.17 (0.98-1.39)	739,620	373	1.10 (0.93-1.30)	1,054,481	571	1.16 (1.01-1.32)
**Albumin**												
Q4	192,426	116	Ref.	478,547	243	Ref.	756,536	280	Ref.	1,090,916	440	Ref.
Q3	191,838	149	1.19 (0.93-1.51)	476,808	275	1.04 (0.87-1.23)	753,153	305	1.02 (0.86-1.20)	1,088,582	447	0.93 (0.81-1.06)
Q2	190,644	166	1.26 (0.99-1.60)	473,421	315	1.12 (0.95-1.33)	746,524	330	1.06 (0.90-1.25)	1,079,346	475	0.94 (0.82-1.07)
Q1	190,738	237	1.71 (1.36-2.15)	472,239	336	1.13 (0.95-1.33)	741,637	355	1.10 (0.94-1.29)	1,068,447	527	0.99 (0.87-1.12)

Models are adjusted for age, sex, BMI, ethnicity, Townsend deprivation index, educational qualifications, physical activity, alcohol consumption, packyears of smoking, intake of vegetables, fruit, fish, whole and refined grains, and red and processed meat, history of CRC screening, family history of CRC, and regular intake of NSAIDs. The cut-offs for the biomarkers were as follows: CRP (0.65, 1.32, 2.74); NLR (1.66, 2.13, 2.76); PLR (105.27, 132.00, 165.83); PNR (48.82, 61.51, 77.33); LMR (3.25, 4.20, 5.33); SII (391.67, 527.40, 713.09); NMLR (1.88, 2.38, 3.05); SIRI (0.68, 0.95, 1.35); and albumin (43.52, 45.22, 46.94). For PNR, LMR, and albumin, the direction of comparison is reversed, and Q4 was used as the reference category; for all other biomarkers, Q1 was used as the reference category.

Table S5. Hazard ratios (95% confidence intervals) of colorectal cancer risk across quartiles of various biomarkers, for cumulative follow-up periods, after excluding extreme biomarker values.

Biomarker/follow-up years included	0–2 (N= 297,367)			0–5 (N= 297,367)			0–8 (N= 297,367)			Complete follow-up (N= 297,367)		
**CRP**	Person-years	n Cases	HR (95% CI)	Person-years	n Cases	HR (95% CI)	Person-years	n Cases	HR (95% CI)	Person-years	n Cases	HR (95% CI)
Q1	145,858	76	Ref.	363,425	250	Ref.	578,097	456	Ref.	847,644	739	Ref.
Q2	150,909	102	1.14 (0.85-1.55)	375,733	308	1.02 (0.86-1.21)	597,033	557	1.00 (0.89-1.14)	873,329	931	1.04 (0.95-1.15)
Q3	147,962	119	1.32 (0.98-1.77)	368,086	363	1.16 (0.98-1.37)	584,284	653	1.13 (1.00-1.28)	852,834	1,027	1.10 (1.00-1.22)
Q4	148,695	177	1.97 (1.47-2.63)	369,332	432	1.35 (1.14-1.60)	585,102	688	1.16 (1.02-1.32)	851,705	1,085	1.13 (1.02-1.25)
**NLR**												
Q1	148,417	96	Ref.	369,511	289	Ref.	587,143	541	Ref.	856,596	864	Ref.
Q2	148,379	114	1.16 (0.88-1.52)	369,436	315	1.06 (0.91-1.25)	586,932	550	0.99 (0.88-1.11)	859,214	906	1.02 (0.93-1.12)
Q3	147,975	116	1.16 (0.88-1.52)	368,142	352	1.17 (1.00-1.37)	584,414	605	1.07 (0.95-1.20)	853,849	966	1.07 (0.98-1.18)
Q4	148,654	148	1.42 (1.10-1.85)	369,486	397	1.27 (1.09-1.48)	586,027	658	1.12 (1.00-1.26)	855,854	1,046	1.13 (1.03-1.24)
**PLR**												
Q1	148,332	111	Ref.	368,787	334	Ref.	584,753	609	Ref.	841,562	976	Ref.
Q2	148,359	120	1.19 (0.91-1.54)	369,189	343	1.12 (0.96-1.30)	586,204	580	1.03 (0.92-1.16)	853,747	950	1.03 (0.95-1.13)
Q3	148,326	109	1.12 (0.86-1.47)	369,230	343	1.16 (1.00-1.35)	586,643	597	1.11 (0.99-1.24)	860,782	973	1.09 (1.00-1.19)
Q4	148,407	134	1.45 (1.12-1.87)	369,369	333	1.18 (1.01-1.38)	586,917	568	1.10 (0.98-1.24)	869,423	883	1.02 (0.93-1.12)
**PNR**												
Q4	148,190	104	Ref.	369,261	258	Ref.	587,423	468	Ref.	870,004	785	Ref.
Q3	148,610	104	0.92 (0.70-1.20)	369,928	339	1.21 (1.03-1.42)	587,831	594	1.16 (1.03-1.32)	862,563	939	1.12 (1.02-1.23)
Q2	148,367	124	1.02 (0.78-1.33)	369,153	357	1.19 (1.01-1.40)	586,134	612	1.12 (0.99-1.27)	853,458	985	1.12 (1.01-1.23)
Q1	148,258	142	1.06 (0.82-1.38)	368,234	399	1.22 (1.04-1.43)	583,129	680	1.14 (1.01-1.29)	839,488	1,073	1.14 (1.03-1.25)
**LMR**												
Q4	148,325	91	Ref.	369,392	280	Ref.	587,142	477	Ref.	857,731	806	Ref.
Q3	148,565	101	1.03 (0.78-1.37)	369,897	315	1.04 (0.89-1.23)	587,763	558	1.08 (0.96-1.22)	860,175	909	1.05 (0.95-1.15)
Q2	150,270	124	1.16 (0.88-1.52)	373,843	339	1.02 (0.87-1.20)	593,482	598	1.06 (0.93-1.19)	867,226	931	0.98 (0.89-1.08)
Q1	146,264	158	1.34 (1.02-1.75)	363,443	419	1.15 (0.98-1.34)	576,129	721	1.16 (1.02-1.31)	840,380	1,136	1.10 (1.00-1.21)
**SII**												
Q1	148,386	97	Ref.	369,324	293	Ref.	586,451	550	Ref.	850,891	892	Ref.
Q2	148,366	101	1.07 (0.81-1.41)	369,337	329	1.15 (0.98-1.34)	586,561	578	1.07 (0.95-1.20)	856,354	942	1.06 (0.97-1.17)
Q3	148,352	136	1.46 (1.12-1.90)	369,071	353	1.25 (1.07-1.45)	586,036	597	1.12 (1.00-1.26)	858,226	973	1.11 (1.01-1.21)
Q4	148,320	140	1.56 (1.20-2.02)	368,844	378	1.38 (1.18-1.61)	585,468	629	1.22 (1.09-1.37)	860,043	975	1.14 (1.04-1.25)
**NMLR**												
Q1	148,402	91	Ref.	369,547	269	Ref.	587,292	510	Ref.	857,038	831	Ref.
Q2	148,486	113	1.20 (0.91-1.59)	369,649	331	1.19 (1.01-1.40)	587,225	572	1.08 (0.96-1.22)	859,253	930	1.08 (0.98-1.18)
Q3	148,172	116	1.21 (0.92-1.59)	368,655	350	1.23 (1.05-1.45)	585,273	607	1.12 (1.00-1.26)	855,405	962	1.10 (1.00-1.21)
Q4	148,365	154	1.53 (1.17-1.99)	368,724	403	1.36 (1.16-1.59)	584,728	665	1.18 (1.05-1.32)	853,817	1,059	1.16 (1.06-1.27)

Biomarker/follow-up years included	0–2 (N= 297,367)			0–5 (N= 297,367)			0–8 (N= 297,367)			Complete follow-up (N= 297,367)		
**SIRI**												
Q1	148,394	85	Ref.	369,801	260	Ref.	588,228	469	Ref.	862,995	789	Ref.
Q2	148,444	103	1.12 (0.84-1.50)	369,558	300	1.06 (0.90-1.26)	587,108	544	1.06 (0.94-1.20)	859,465	873	1.02 (0.93-1.13)
Q3	148,325	114	1.17 (0.88-1.55)	369,052	355	1.18 (1.00-1.39)	585,978	607	1.11 (0.98-1.25)	855,684	966	1.07 (0.97-1.17)
Q4	148,260	172	1.57 (1.20-2.06)	368,164	438	1.30 (1.11-1.52)	583,202	734	1.20 (1.06-1.35)	847,369	1,154	1.16 (1.05-1.27)
**Albumin**												
Q4	148,709	92	Ref.	370,333	279	Ref.	588,649	511	Ref.	857,092	840	Ref.
Q3	149,018	108	1.07 (0.81-1.42)	370,964	306	1.00 (0.85-1.18)	589,338	545	0.98 (0.87-1.11)	861,235	874	0.95 (0.87-1.05)
Q2	147,638	117	1.12 (0.85-1.47)	367,306	353	1.11 (0.94-1.30)	583,014	602	1.04 (0.92-1.17)	852,981	965	1.01 (0.92-1.11)
Q1	148,059	157	1.41 (1.08-1.83)	367,972	415	1.22 (1.04-1.42)	583,516	696	1.13 (1.01-1.27)	854,205	1,103	1.08 (0.99-1.19)

Models are adjusted for age, sex, BMI, ethnicity, Townsend deprivation index, educational qualifications, physical activity, alcohol consumption, packyears of smoking, intake of vegetables, fruit, fish, whole and refined grains, and red and processed meat, history of CRC screening, family history of CRC, and regular intake of NSAIDs. The cut-offs for the biomarkers were as follows: CRP (0.60, 1.16, 2.16); NLR (1.66, 2.07, 2.58); PLR (103.72, 128.27, 157.62); PNR (50.38, 62.29, 76.67); LMR (3.40, 4.25, 5.28); SII (383.87, 503.76, 653.18); NMLR (1.88, 2.32, 2.86); SIRI (0.69, 0.93, 1.25); and albumin (43.70, 45.34, 47.02). For PNR, LMR, and albumin, the direction of comparison was reversed, and Q4 was used as the reference category; for all other biomarkers, Q1 was used as the reference category.

Table S6. Hazard ratios (95% confidence intervals) of colorectal cancer risk across quartiles of various biomarkers, during follow-up years 0–2, 3–5, 6–8, and beyond 8 years, after excluding extreme biomarker values.

Biomarker/follow-up years included	0–2 (N=297,367)			3–5 (N=296,009)			6–8 (N=292,425)			>8 (N=285,958)		
**CRP**	Person-years	n Cases	HR (95% CI)	Person-years	n Cases	HR (95% CI)	Person-years	n Cases	HR (95% CI)	Person-years	n Cases	HR (95% CI)
Q1	145,858	76	Ref.	363,168	174	Ref.	575,308	206	Ref.	837,129	283	Ref.
Q2	150,909	102	1.14 (0.85-1.55)	375,391	206	0.96 (0.78-1.18)	593,855	249	0.99 (0.82-1.19)	860,076	374	1.11 (0.95-1.29)
Q3	147,962	119	1.32 (0.98-1.77)	367,686	244	1.08 (0.89-1.32)	580,537	290	1.09 (0.91-1.32)	837,886	374	1.06 (0.90-1.24)
Q4	148,695	177	1.97 (1.47-2.63)	368,756	255	1.10 (0.89-1.35)	580,285	256	0.93 (0.76-1.13)	833,563	397	1.08 (0.91-1.28)
**NLR**												
Q1	148,417	96	Ref.	369,172	193	Ref.	583,909	252	Ref.	843,885	323	Ref.
Q2	148,379	114	1.16 (0.88-1.52)	369,113	201	1.02 (0.83-1.24)	583,666	235	0.90 (0.75-1.08)	846,023	356	1.07 (0.92-1.24)
Q3	147,975	116	1.16 (0.88-1.52)	367,735	236	1.17 (0.97-1.42)	580,632	253	0.95 (0.80-1.14)	839,059	361	1.08 (0.93-1.26)
Q4	148,654	148	1.42 (1.10-1.85)	368,982	249	1.20 (0.99-1.45)	581,778	261	0.95 (0.80-1.13)	839,686	388	1.14 (0.98-1.32)
**PLR**												
Q1	148,332	111	Ref.	368,342	223	Ref.	580,416	275	Ref.	825,004	367	Ref.
Q2	148,359	120	1.19 (0.91-1.54)	368,822	223	1.08 (0.90-1.31)	582,603	237	0.93 (0.78-1.11)	839,343	370	1.03 (0.89-1.19)
Q3	148,326	109	1.12 (0.86-1.47)	368,872	234	1.18 (0.98-1.42)	583,413	254	1.04 (0.87-1.23)	847,659	376	1.05 (0.91-1.21)
Q4	148,407	134	1.45 (1.12-1.87)	368,966	199	1.05 (0.87-1.28)	583,553	235	1.01 (0.84-1.20)	856,647	315	0.89 (0.77-1.04)
**PNR**												
Q4	148,190	104	Ref.	368,974	154	Ref.	584,750	210	Ref.	859,363	317	Ref.
Q3	148,610	104	0.92 (0.70-1.20)	369,563	235	1.40 (1.15-1.72)	584,539	255	1.11 (0.92-1.33)	849,780	345	1.05 (0.90-1.22)
Q2	148,367	124	1.02 (0.78-1.33)	368,779	233	1.31 (1.06-1.61)	582,449	255	1.04 (0.86-1.25)	839,101	373	1.11 (0.95-1.29)
Q1	148,258	142	1.06 (0.82-1.38)	367,686	257	1.32 (1.08-1.63)	578,247	281	1.05 (0.87-1.26)	820,410	393	1.14 (0.97-1.32)
**LMR**												
Q4	148,325	91	Ref.	369,084	189	Ref.	584,004	197	Ref.	845,733	329	Ref.
Q3	148,565	101	1.03 (0.78-1.37)	369,549	214	1.05 (0.86-1.28)	584,600	243	1.14 (0.94-1.37)	847,132	351	1.00 (0.86-1.16)
Q2	150,270	124	1.16 (0.88-1.52)	373,438	215	0.96 (0.78-1.17)	589,684	259	1.10 (0.91-1.33)	852,550	333	0.88 (0.75-1.03)
Q1	146,264	158	1.34 (1.02-1.75)	362,931	261	1.05 (0.87-1.28)	571,697	302	1.17 (0.97-1.41)	823,239	415	1.03 (0.88-1.19)
**SII**												
Q1	148,386	97	Ref.	368,971	196	Ref.	583,029	257	Ref.	836,771	342	Ref.
Q2	148,366	101	1.07 (0.81-1.41)	368,995	228	1.18 (0.98-1.43)	583,058	249	0.98 (0.82-1.17)	842,398	364	1.06 (0.91-1.22)
Q3	148,352	136	1.46 (1.12-1.90)	368,656	217	1.14 (0.94-1.38)	582,322	244	0.97 (0.82-1.16)	844,254	376	1.09 (0.94-1.26)
Q4	148,320	140	1.56 (1.20-2.02)	368,379	238	1.29 (1.07-1.56)	581,577	251	1.04 (0.87-1.24)	845,230	346	1.02 (0.88-1.19)
**NMLR**												
Q1	148,402	91	Ref.	369,222	178	Ref.	584,142	241	Ref.	844,570	321	Ref.
Q2	148,486	113	1.20 (0.91-1.59)	369,319	218	1.18 (0.97-1.45)	583,896	241	0.96 (0.80-1.15)	845,962	358	1.07 (0.92-1.25)
Q3	148,172	116	1.21 (0.92-1.59)	368,253	234	1.25 (1.02-1.51)	581,553	257	1.00 (0.84-1.19)	840,724	355	1.06 (0.91-1.23)
Q4	148,365	154	1.53 (1.17-1.99)	368,208	249	1.27 (1.04-1.54)	580,395	262	0.97 (0.82-1.16)	837,397	394	1.14 (0.98-1.32)

Biomarker/follow-up years included	0–2 (N=297,367)			3–5 (N=296,009)			6–8 (N=292,425)			>8 (N=285,958)		
**SIRI**												
Q1	148,394	85	Ref.	369,541	175	Ref.	585,550	209	Ref.	852,111	320	Ref.
Q2	148,444	103	1.12 (0.84-1.50)	369,219	197	1.03 (0.84-1.27)	583,810	244	1.06 (0.88-1.28)	846,395	329	0.97 (0.83-1.13)
Q3	148,325	114	1.17 (0.88-1.55)	368,653	241	1.18 (0.97-1.44)	582,316	252	1.02 (0.85-1.23)	841,250	359	1.01 (0.86-1.17)
Q4	148,260	172	1.57 (1.20-2.06)	367,589	266	1.17 (0.96-1.42)	578,308	296	1.07 (0.89-1.28)	828,899	420	1.10 (0.94-1.28)
**Albumin**												
Q4	148,709	92	Ref.	370,021	187	Ref.	585,537	232	Ref.	844,951	329	Ref.
Q3	149,018	108	1.07 (0.81-1.42)	370,604	198	0.97 (0.79-1.18)	586,064	239	0.95 (0.80-1.14)	847,711	329	0.91 (0.78-1.06)
Q2	147,638	117	1.12 (0.85-1.47)	366,906	236	1.10 (0.91-1.33)	579,209	249	0.96 (0.80-1.15)	838,310	363	0.96 (0.82-1.11)
Q1	148,059	157	1.41 (1.08-1.83)	367,470	258	1.12 (0.93-1.36)	579,175	281	1.03 (0.86-1.23)	837,682	407	1.00 (0.86-1.16)

Models are adjusted for age, sex, BMI, ethnicity, Townsend deprivation index, educational qualifications, physical activity, alcohol consumption, packyears of smoking, intake of vegetables, fruit, fish, whole and refined grains, and red and processed meat, history of CRC screening, family history of CRC, and regular intake of NSAIDs. The cut-offs for the biomarkers were as follows: CRP (0.60, 1.16, 2.16); NLR (1.66, 2.07, 2.58); PLR (103.72, 128.27, 157.62); PNR (50.38, 62.29, 76.67); LMR (3.40, 4.25, 5.28); SII (383.87, 503.76, 653.18); NMLR (1.88, 2.32, 2.86); SIRI (0.69, 0.93, 1.25); and albumin (43.70, 45.34, 47.02). For PNR, LMR, and albumin, the direction of comparison was reversed, and Q4 was used as the reference category; for all other biomarkers, Q1 was used as the reference category.

Table S7. Associations between baseline C-reactive protein and colorectal cancer risk, for cumulative follow-up periods, according to, sex and cancer site, in the CRP-specific complete-case population.

CRP measure/follow-up years included	0–2 (N=430,871)		0–5 (N=430,871)		0–8 (N=430,871)		Complete follow-up (N=430,871)	
**Overall**	**n Cases**	**HR (95% CI)**	**n Cases**	**HR (95% CI)**	**n Cases**	**HR (95% CI)**	**n Cases**	**HR (95% CI)**
**CRP quartiles**								
Q1 (reference)	106	Ref.	360	Ref.	651	Ref.	1,079	Ref.
Q2	155	1.28 (1.00-1.65)	466	1.10 (0.96-1.27)	819	1.07 (0.96-1.18)	1,358	1.07 (0.99-1.16)
Q3	197	1.57 (1.23-2.01)	559	1.24 (1.08-1.42)	951	1.16 (1.04-1.28)	1,513	1.11 (1.03-1.21)
Q4	280	2.33 (1.83-2.96)	667	1.48 (1.29-1.71)	1,030	1.25 (1.12-1.39)	1,618	1.18 (1.09-1.29)
Ln (CRP)	738	1.33 (1.24-1.43)	2,052	1.15 (1.10-1.20)	3,451	1.09 (1.05-1.13)	5,568	1.07 (1.04-1.10)
Per 1 SD increase	738	1.35 (1.25-1.46)	2,052	1.16 (1.11-1.22)	3,451	1.09 (1.05-1.14)	5,568	1.07 (1.04-1.10)
**Men**								
CRP quartiles								
Q1 (reference)	57	Ref.	192	Ref.	353	Ref.	584	Ref.
Q2	95	1.34 (0.96-1.87)	279	1.14 (0.95-1.37)	498	1.11 (0.97-1.27)	795	1.08 (0.97-1.20)
Q3	115	1.56 (1.12-2.25)	332	1.28 (1.07-1.54)	587	1.23 (1.07-1.41)	889	1.14 (1.02-1.27)
Q4	184	2.73 (1.99-3.74)	398	1.67 (1.39-2.00)	600	1.37 (1.19-1.57)	919	1.29 (1.15-1.44)
**Women**								
CRP quartiles								
Q1 (reference)	49	Ref.	168	Ref.	298	Ref.	495	Ref.
Q2	60	1.21 (0.83-1.78)	187	1.06 (0.86-1.31)	321	1.02 (0.87-1.20)	563	1.07 (0.95-1.21)
Q3	82	1.60 (1.10-2.31)	227	1.19 (0.96-1.46)	364	1.06 (0.90-1.25)	624	1.09 (0.96-1.23)
Q4	96	1.83 (1.25-2.68)	269	1.28 (1.03-1.59)	430	1.12 (0.95-1.32)	699	1.07 (0.94-1.22)
**Colon cancer**								
CRP quartiles								
Q1 (reference)	65	Ref.	230	Ref.	419	Ref.	704	Ref.
Q2	91	1.22 (0.88-1.68)	297	1.10 (0.92-1.31)	517	1.04 (0.91-1.18)	894	1.07 (0.97-1.19)
Q3	132	1.69 (1.24-2.29)	394	1.36 (1.15-1.61)	658	1.22 (1.08-1.39)	1,048	1.16 (1.05-1.28)
Q4	194	2.54 (1.87-3.43)	462	1.58 (1.33-1.87)	695	1.26 (1.10-1.44)	1,102	1.19 (1.07-1.32)
**Rectal cancer**								
CRP quartiles								
Q1 (reference)	42	Ref.	133	Ref.	237	Ref.	379	Ref.
Q2	67	1.41 (0.95-2.08)	173	1.11 (0.88-1.39)	307	1.11 (0.94-1.32)	466	1.07 (0.93-1.23)
Q3	68	1.39 (0.94-2.08)	169	1.03 (0.81-1.30)	300	1.04 (0.87-1.24)	473	1.03 (0.90-1.19)
Q4	88	1.93 (1.30-2.87)	209	1.31 (1.03-1.66)	342	1.23 (1.03-1.48)	521	1.18 (1.02-1.36)

*Analyses included all participants with available CRP measurements, irrespective of missing values for other inflammatory biomarkers.

Models are adjusted for age, sex, BMI, ethnicity, Townsend deprivation index, educational qualifications, physical activity, alcohol consumption, packyears of smoking, intake of vegetables, fruit, fish, whole and refined grains, and red and processed meat, history of CRC screening, family history of CRC, and regular intake of NSAIDs. The CRP cut-offs were 0.65, 1.31, and 2.72 mg/L.

Table S8. Associations between baseline C-reactive protein and colorectal cancer risk, during follow-up years 0–2, 3–5, 6–8, and beyond 8 years, according to, sex and cancer site, in the CRP-specific complete-case population.

CRP measure/follow-up years included	0–2 (N=430,871)		3–5 (N= 428,495)		6–8 (N=422,518)		>8 (N=412,309)	
**Overall**	**n Cases**	**HR (95% CI)**	**n Cases**	**HR (95% CI)**	**n Cases**	**HR (95% CI)**	**n Cases**	**HR (95% CI)**
**CRP quartiles**								
Q1 (reference)	106	Ref.	254	Ref.	291	Ref.	428	Ref.
Q2	155	1.28 (1.00-1.65)	311	1.02 (0.87-1.21)	353	1.02 (0.87-1.20)	539	1.08 (0.95-1.23)
Q3	197	1.57 (1.23-2.01)	362	1.10 (0.93-1.30)	392	1.05 (0.90-1.23)	562	1.05 (0.92-1.20)
Q4	280	2.33 (1.83-2.96)	387	1.15 (0.97-1.37)	363	0.96 (0.81-1.14)	588	1.08 (0.94-1.24)
Ln (CRP)	738	1.33 (1.24-1.43)	1,314	1.05 (0.99-1.12)	1,399	1.00 (0.95-1.06)	2,117	1.03 (0.98-1.08)
Per 1 SD increase	738	1.35 (1.25-1.46)	1,314	1.06 (0.99-1.12)	1,399	1.00 (0.94-1.06)	2,117	1.03 (0.98-1.08)
**Men**								
CRP quartiles								
Q1 (reference)	57	Ref.	135	Ref.	161	Ref.	231	Ref.
Q2	95	1.34 (0.96-1.87)	184	1.05 (0.84-1.32)	219	1.07 (0.87-1.32)	297	1.03 (0.87-1.23)
Q3	115	1.56 (1.12-2.25)	217	1.16 (0.93-1.45)	255	1.17 (0.95-1.44)	302	0.99 (0.83-1.18)
Q4	184	2.73 (1.99-3.74)	214	1.23 (0.98-1.55)	202	1.01 (0.81-1.26)	319	1.17 (0.97-1.40)
**Women**								
CRP quartiles								
Q1 (reference)	49	Ref.	119	Ref.	130	Ref.	197	Ref.
Q2	60	1.21 (0.83-1.78)	127	0.99 (0.77-1.28)	134	0.97 (0.76-1.23)	242	1.16 (0.96-1.40)
Q3	82	1.60 (1.10-2.31)	145	1.03 (0.80-1.32)	137	0.90 (0.70-1.16)	260	1.12 (0.92-1.37)
Q4	96	1.83 (1.25-2.68)	173	1.07 (0.82-1.39)	161	0.92 (0.70-1.20)	269	1.00 (0.81-1.24)
**Colon cancer**								
CRP quartiles								
Q1 (reference)	65	Ref.	165	Ref.	189	Ref.	285	Ref.
Q2	91	1.22 (0.88-1.68)	206	1.05 (0.85-1.29)	220	0.96 (0.79-1.17)	377	1.12 (0.96-1.31)
Q3	132	1.69 (1.24-2.29)	262	1.22 (1.00-1.50)	264	1.05 (0.87-1.28)	390	1.07 (0.91-1.25)
Q4	194	2.54 (1.87-3.43)	268	1.21 (0.98-1.50)	233	0.88 (0.72-1.09)	407	1.08 (0.92-1.28)
**Rectal cancer**								
CRP quartiles								
Q1 (reference)	42	Ref.	91	Ref.	104	Ref.	142	Ref.
Q2	67	1.41 (0.95-2.08)	106	0.97 (0.73-1.29)	134	1.13 (0.87-1.46)	159	0.99 (0.79-1.25)
Q3	68	1.39 (0.94-2.08)	101	0.86 (0.64-1.15)	131	1.06 (0.81-1.38)	173	1.02 (0.81-1.29)
Q4	88	1.93 (1.30-2.87)	121	1.04 (0.77-1.40)	133	1.12 (0.85-1.49)	179	1.08 (0.85-1.38)

*Analyses included all participants with available CRP measurements, irrespective of missing values for other inflammatory biomarkers.

Models are adjusted for age, sex, BMI, ethnicity, Townsend deprivation index, educational qualifications, physical activity, alcohol consumption, packyears of smoking, intake of vegetables, fruit, fish, whole and refined grains, and red and processed meat, history of CRC screening, family history of CRC, and regular intake of NSAIDs. The CRP cut-offs were 0.65, 1.31, and 2.72 mg/L.

Table S9. Associations between baseline C-reactive protein and colorectal cancer risk, during various follow-up periods, in the CRP-specific complete-case population, after excluding participants with prevalent inflammatory bowel disease (IBD).

**CRP measure/follow-up years included**	**0–2 (N= 419,987)**		**0–5 (N= 419,987)**		**0–8 (N= 419,987)**		**Complete follow-up (N= 419,987)**	
	**n Cases**	**HR (95% CI)**	**n Cases**	**HR (95% CI)**	**n Cases**	**HR (95% CI)**	**n Cases**	**HR (95% CI)**
**CRP quartiles**								
Q1 (reference)	104	Ref.	355	Ref.	640	Ref.	1,061	Ref.
Q2	151	1.27 (0.99-1.64)	454	1.09 (0.95-1.25)	800	1.06 (0.96-1.18)	1,332	1.07 (0.99-1.16)
Q3	192	1.56 (1.22-2.00)	549	1.24 (1.08-1.42)	927	1.15 (1.04-1.28)	1,468	1.10 (1.02-1.20)
Q4	271	2.30 (1.80-2.94)	642	1.46 (1.27-1.68)	994	1.24 (1.11-1.38)	1,565	1.18 (1.08-1.28)
	**0–2 (N=419,987)**		**3–5 (N=417,714)**		**6–8 (N=411,958)**		**>8 (N=402,136)**	
	**n Cases**	**HR (95% CI)**	**n Cases**	**HR (95% CI)**	**n Cases**	**HR (95% CI)**	**n Cases**	**HR (95% CI)**
**CRP quartiles**								
Q1 (reference)	104	Ref.	251	Ref.	285	Ref.	421	Ref.
Q2	151	1.27 (0.99-1.64)	303	1.01 (0.85-1.20)	346	1.03 (0.88-1.20)	532	1.09 (0.96-1.24)
Q3	192	1.56 (1.22-2.00)	357	1.10 (0.93-1.30)	378	1.04 (0.89-1.23)	541	1.03 (0.90-1.18)
Q4	271	2.30 (1.80-2.94)	371	1.13 (0.95-1.35)	352	0.97 (0.81-1.15)	571	1.08 (0.94-1.24)

*Analyses included all participants with available CRP measurements, irrespective of missing values for other inflammatory biomarkers.

Models are adjusted for age, sex, BMI, ethnicity, Townsend deprivation index, educational qualifications, physical activity, alcohol consumption, packyears of smoking, intake of vegetables, fruit, fish, whole and refined grains, and red and processed meat, history of CRC screening, family history of CRC, and regular intake of NSAIDs. The CRP cut-offs were 0.65, 1,31, and 2.72 mg/L.

Table S10. Fine–Gray competing-risk analysis of the associations between C-reactive protein and colorectal cancer risk across cumulative follow-up periods in the CRP-specific complete-case population[a].

**CRP measure/follow-up years included**	**0–1 (N=430,871)**		**0–2 (N=430,871)**		**0–5 (N=430,871)**		**0–8 (N=430,871)**		**Complete follow-up (N=430,871)**	
	**n Cases**	**SHR (95% CI)[b]**	**n Cases**	**SHR (95% CI)[b]**	**n Cases**	**SHR (95% CI)[b]**	**n Cases**	**SHR (95% CI)[b]**	**n Cases**	**SHR (95% CI)[b]**
**CRP quartiles**										
Q1 (reference)	41	Ref.	106	Ref.	360	Ref.	651	Ref.	1,079	Ref.
Q2	63	1.35 (0.90-2.01)	155	1.28 (1.00-1.65)	466	1.10 (0.96-1.27)	819	1.07 (0.96-1.18)	1,358	1.07 (0.99-1.17)
Q3	90	1.87 (1.27-2.75)	197	1.57 (1.23-2.01)	559	1.24 (1.08-1.42)	951	1.16 (1.04-1.28)	1,513	1.11 (1.03-1.21)
Q4	143	3.13 (2.14-4.57)	280	2.32 (1.81-2.96)	667	1.47 (1.28-1.70)	1,030	1.24 (1.11-1.38)	1,618	1.16 (1.07-1.27)

[a]Analyses included all participants with available CRP measurements, irrespective of missing values for other inflammatory biomarkers.
[b]Death before CRC diagnosis was treated as the competing event. Estimates are presented as subdistribution hazard ratios (SHRs) with 95% confidence intervals.

Models are adjusted for age, sex, BMI, ethnicity, Townsend deprivation index, educational qualifications, physical activity, alcohol consumption, packyears of smoking, intake of vegetables, fruit, fish, whole and refined grains, and red and processed meat, history of CRC screening, family history of CRC, and regular intake of NSAIDs. The CRP cut-offs were 0.65, 1,31, and 2.72 mg/L.

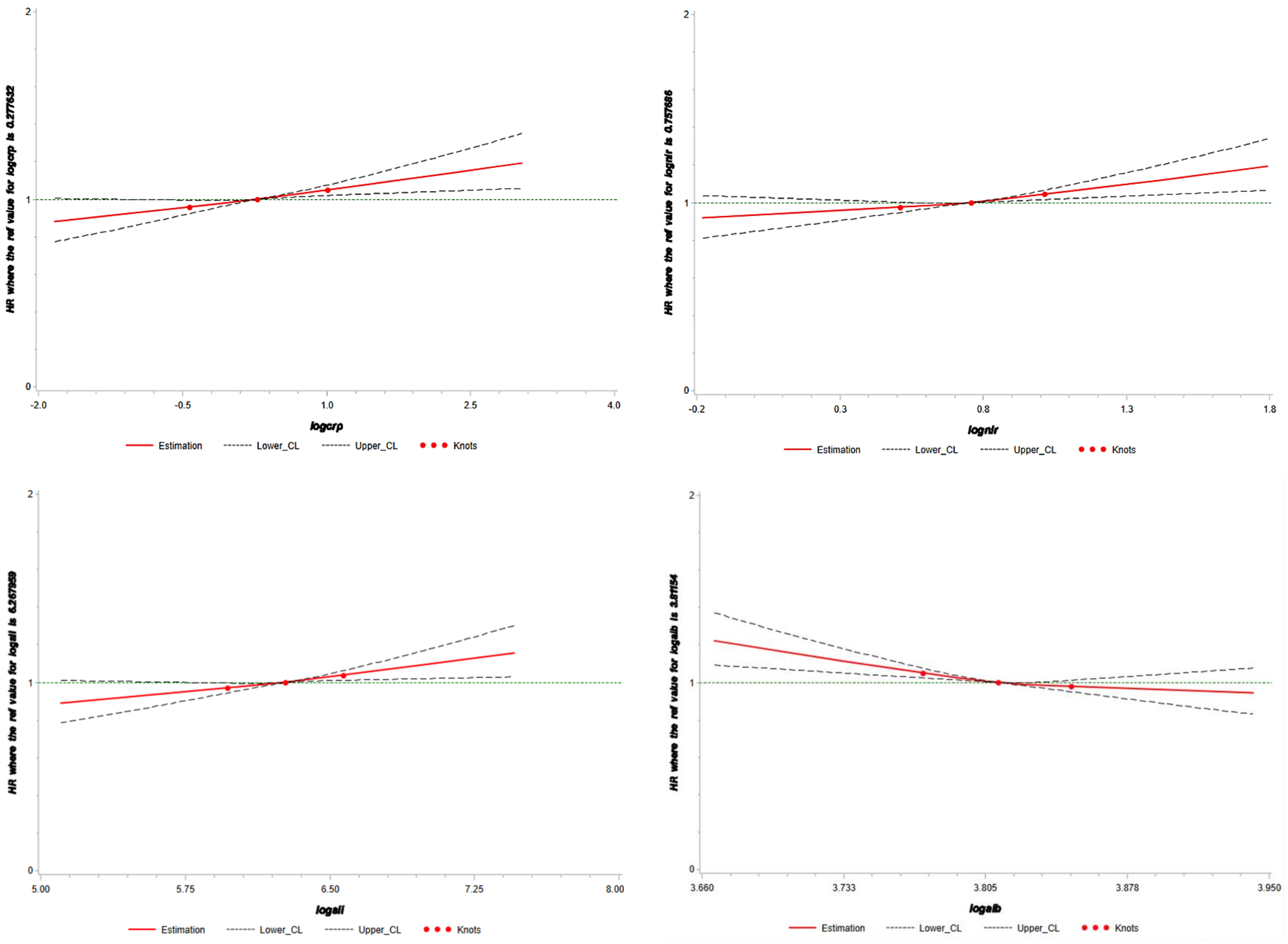

Figure S1. Restricted cubic spline analyses of the associations between inflammatory biomarkers and colorectal cancer risk. The plots show hazard ratios (HRs) and corresponding 95% confidence intervals (CIs) across the continuous distributions of the natural logarithm of CRP, NLR, SII, albumin. The median value of each biomarker was used as the reference value.
